# Ruminant Species Composition and Reported Methane Intensity in Global Milk Production: A 186-Country Decomposition Under Alternative Allocation Boundaries

**DOI:** 10.3390/ani16203273

**Published:** 2026-10-17

**Authors:** Otto Ketney

**Affiliations:** Department of Agricultural Science and Food Engineering, Lucian Blaga University of Sibiu, 7–9 Dr. I. Ratiu Street, 550012 Sibiu, Romania; otto.ketney@ulbsibiu.ro

**Keywords:** ruminant species, milk methane intensity, allocation boundary, FAOSTAT Tier 1, Shapley decomposition, functional unit, influential observations, herd function

## Abstract

Methane intensity of milk is often compared across ruminant species, but the Food and Agriculture Organization Corporate Statistical Database (FAOSTAT) identifies a dairy herd only for cattle; for buffalo, goats, sheep and camels it reports whole-herd methane. For 186 countries in 2020–2023 we allocated non-bovine herd methane to milk using country-specific shares of animals in milk. This reduced pooled intensity ratios to cattle from 2.9–17.9 to 1.1–5.2, and cattle had the lowest reported milk-allocated intensity in 53 of 107 multispecies countries (89 under whole-herd allocation). In the screened primary analysis (one implausible Mongolian sheep-milk series excluded), the world ratio decreased by 0.64 g CH_4_ kg^−1^; the three-factor decomposition attributed +0.48 g CH_4_ kg^−1^ to country shares, −0.10 to species mix and −1.02 to within-species intensity. With the series retained, the corresponding components were +0.57, −1.43 and +0.21. Cross-species comparisons are accounting constructs until boundaries and influential records are explicit.

## Simple Summary

Milk comes from dairy cattle, but also from buffalo, goats, sheep and camels, and countries are often compared by the methane released per kilogram of milk. For cattle, national statistics count only dairy cows; for the other species they count whole herds, including animals kept for meat or wool. We recalculated the comparison for 186 countries in 2020–2023, charging each species’ methane to milk only in proportion to the animals that are milked. This narrowed the gap between cattle and the other species several-fold: cattle had the lowest reported milk-allocated methane intensity in 53 of the 107 countries that milk more than one species, against 89 when whole herds were counted. Worldwide methane per kilogram of milk fell slightly. One implausible Mongolian record strongly affected attribution of that change; the screened and full-panel results are therefore reported separately. Comparisons between milk species depend strongly on how emissions are counted.

## 1. Introduction

Milk from cattle, buffalo, goats, sheep and camels supplies protein and micronutrients to human populations and sustains rural livelihoods in very different production systems [1,2]. Agricultural methane is a priority target for limiting warming to 1.5 °C [3], and the sector is conventionally evaluated through methane per unit of product rather than per animal or per hectare. Because that unit is a ratio, a national figure is by construction a production-weighted average over the species a country milks: the same metric changes when animals become more efficient, and also when the mixture of species supplying the milk changes.

Cattle supply 81% of the raw milk in the analytical panel of this study, but buffalo supply 15.5%, goats 2.1%, sheep 1.0% and camels 0.4% (2023; Section 3.1), and these shares are much larger in South Asia, the Mediterranean, the Sahel and the Horn of Africa. A small share of global volume can therefore be a large share of national milk, and a national intensity cannot be interpreted without knowing which species supply it.

National and global comparisons of that metric depend heavily on how the inventory is built. The Food and Agriculture Organization Corporate Statistical Database (FAOSTAT) estimates livestock methane at Intergovernmental Panel on Climate Change (IPCC) Tier 1, multiplying head counts by default emission factors that are fixed by region and animal category [4,5,6]. Tier 1 factors can understate emissions in some systems [7] and may overstate them in others; for example, measured emissions of indigenous nitrogen-efficient bovids were well below IPCC Tier 2 model predictions [8]. Default factors may therefore either under- or over-represent locally adapted production systems, depending on breed, environment and inventory specification [9,10,11,12]. A second feature is more specific to milk. FAOSTAT resolves cattle into dairy and non-dairy categories, so the cattle numerator is already the methane of the milk cows, but it provides no such split for buffalo, goats, sheep and camels. Their herd methane covers males, young stock and animals kept mainly for meat, fibre, hides or draught, and the FAOSTAT Emissions intensities domain corrects for this by scaling non-bovine emissions with the share of animals producing milk [13].

The functional unit matters for the same reason. Raw milk differs strongly between species in fat, protein and solids, and fat- and protein-corrected milk (FPCM) is the usual remedy [14,15,16]; co-product allocation between milk and meat in turn governs the outcome of dairy carbon-footprint comparisons whenever the meat-to-milk ratio is large [17,18,19,20]. Non-bovine milk species are kept in multi-purpose systems that differ from specialised dairy cattle in body size, individual yield, lactation pattern, forage quality and herd function [21,22,23,24], yet they are rarely analysed in the emission literature, which remains overwhelmingly bovine [25,26,27,28]. As a result, it is unclear how much of the reported difference between species reflects the animals and how much reflects the accounting boundary, and how much a decomposition of national intensity into a species-mix and a within-species term depends on that boundary and on a few influential national records.

Index decomposition is a standard tool of energy and environmental accounting, where exact Shapley-type attribution gives a symmetric split of a change among its drivers [29,30,31,32]. Applied to a mixture indicator such as national milk intensity, it separates the change in species shares from the change in species intensities. Exactness, however, does not make the components robust. When the denominator of a single country–species cell collapses, the change in its share and the change in its intensity are the same event, and the decomposition divides it between two large terms of opposite sign. Influence diagnostics of the kind used in regression [33] are rarely reported next to such decompositions, although they decide whether the attribution is a finding or a property of one record.

Country-level studies show how much emission intensity varies within a species: with the degree of intensification in India [34], between agro-ecological zones in Pakistan [25], in smallholder systems of West Africa [35] and in the national inventory of Egypt, which separates cattle, buffalo, sheep and goats [36]. Global models such as the Global Livestock Environmental Assessment Model (GLEAM) use feed rations and herd structure to estimate emissions [37,38], but FAOSTAT, which provides globally harmonised coverage of the five milk-producing species under a common accounting framework, relies on the Tier 1 approach. Comparisons among species and the decomposition of national trends are therefore, in practice, comparisons of Tier 1 accounting, and their interpretation requires knowing what that accounting does and does not contain. Within the literature surveyed we found no global analysis that combined the allocation of non-bovine herd methane to milk, an exact decomposition and an influence analysis; the survey was not systematic, and no claim of priority is made.

This study quantifies how changes in national ruminant milk-species composition contribute to reported methane intensity and tests whether this attribution persists when methane from non-bovine herds is allocated to milk using country-specific FAOSTAT milk-animal shares. We further evaluate its sensitivity to influential observations and to alternative milk functional units.

## 2. Materials and Methods

### 2.1. Data Sources, Variables and Study Period

Methane, livestock numbers and milk production were taken from two FAOSTAT domains: Emissions from Livestock (GLE) and Crops and Livestock Products (QCL) [4,13]. All variables are listed in Table 1. Milk production was not used to predict methane. Methane emissions were obtained independently from the GLE domain, where they are computed as head counts multiplied by Tier 1 emission factors; milk production entered only as the functional-unit denominator of the intensity indicator, and the milk-animal and stock counts entered only through the allocation factor defined in Section 2.2 and, as the activity data *N* of the Tier 1 diagnostic, in Equations (4) and (5). Methane is reported as CH_4_ mass (kt); no global-warming-potential conversion was applied, so the results do not depend on the choice of global warming potential (GWP) metric, GWP_100_ or GWP*.

In GLE, enteric and manure-management methane are computed at IPCC Tier 1 as the product of the number of animals in a category and a default emission factor that depends on the livestock category, the region and, for some categories, the mean annual temperature of the country [4,5]. Livestock numbers come from the QCL domain, with harmonisation of the dairy and non-dairy cattle categories, and the QCL element ‘Milk animals’ counts the animals in milk. FAOSTAT flags every value as official (A), estimated (E), imputed (I) or from an external organisation (X). Among the non-bovine cells of the analytical panel, 43% of the milk values are official, 10% estimated and 44% imputed, while the milk-animal counts are official in 33%, estimated in 11% and imputed in 56% of the cells. All methane values are flagged as estimated, as expected for Tier 1.

The analysis ends in 2023: milk and stock series extend to 2024, but GLE methane at Tier 1 ends in 2023 (later years in the domain are projections) [4], and the 2020 values of some countries are duplicated by records sourced from the United Nations Framework Convention on Climate Change (UNFCCC); these duplicates were discarded in favour of the FAO Tier 1 series used for all other years. The observation unit is the country–species–year cell. Regional aggregates and the ‘China’ aggregate (United Nations M49 code 159) were removed, retaining ‘China, mainland’ (M49 code 156). The start year is not imposed by data availability, because the QCL and GLE series begin in 1961; the four years 2020–2023 were chosen to describe the most recent four years of the Tier 1 inventory, so that trends are interval comparisons. Supplementary Table S11 repeats the aggregate decomposition for windows beginning in 2015 and 2010 and gives the same attribution (Section 3.5). “Ruminant” is used in this paper in the broad livestock sense for the five FAOSTAT milk species; camels are pseudo-ruminant foregut fermenters (suborder Tylopoda), which FAOSTAT inventories under the same Tier 1 enteric-fermentation approach.

The extraction was repeated directly from the public FAOSTAT bulk files, with the items, elements, sources and filters specified in Supplementary Section S1. Reconstructed values of milk and methane match those of a previous extraction of the same data in all 1617 country–species–year cells shared by the two extractions (maximum relative difference below 10^−15^). The re-extraction also recovered five reporting entities that were absent from the previous extraction (Saudi Arabia, the Syrian Arab Republic, the United Arab Emirates, the Central African Republic and South Africa). FAOSTAT flags and notes of every component are retained in the extracted table, and the stored intensity is named explicitly in g CH_4_ kg^−1^ raw milk.

### 2.2. Accounting Boundaries and Indicators

**Figure 1:**
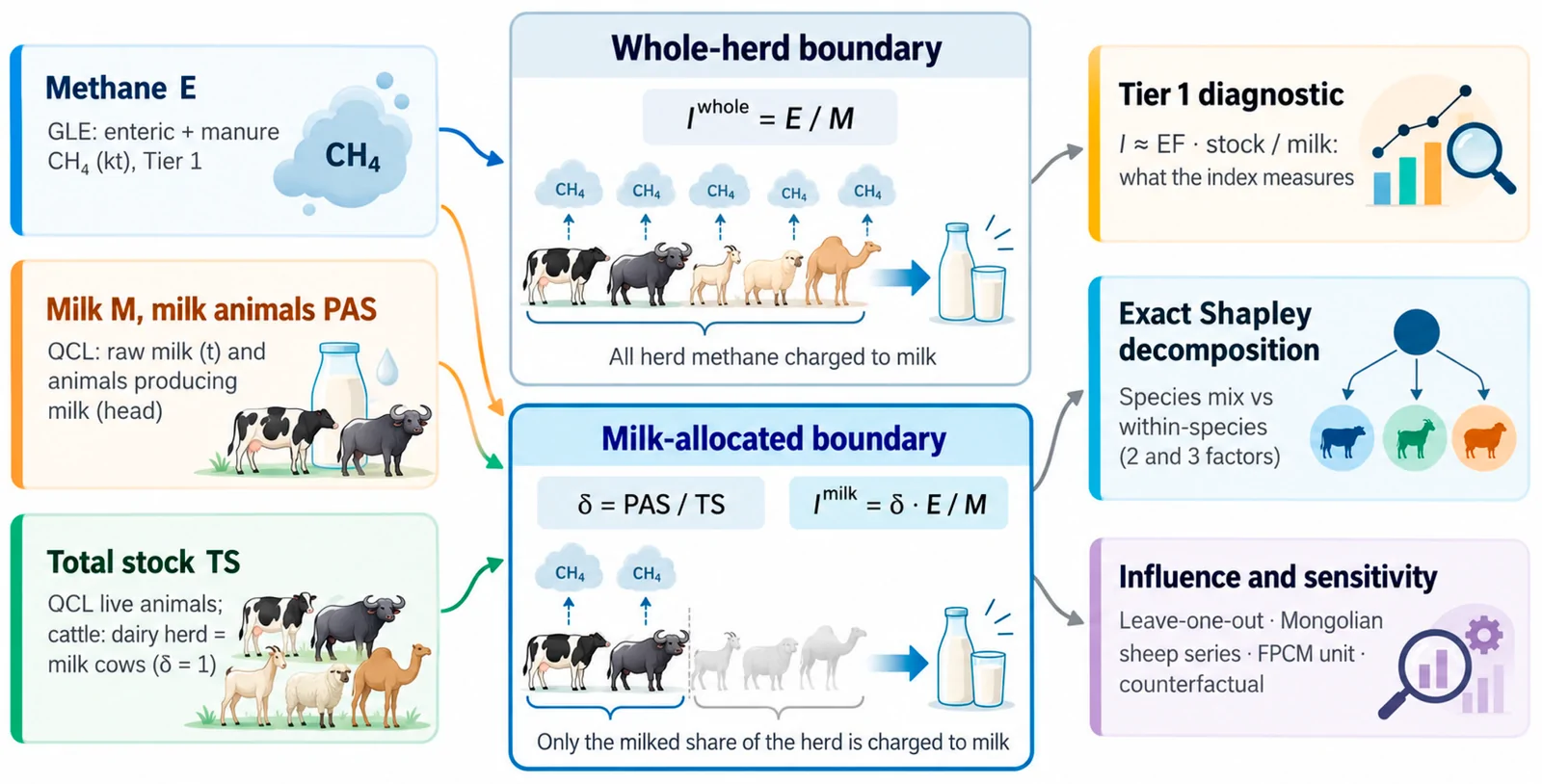
Accounting architecture of the study. FAOSTAT methane, milk, milk-animal and stock series are combined into whole-herd and milk-allocated intensity indicators. For buffalo, goats, sheep and camels, the milk-allocation factor is the country- and year-specific ratio of animals producing milk to QCL total stock (*δ* = *PAS*/*TS*). For cattle, *δ* = 1 because the GLE category “Cattle, dairy” already represents the dairy-cattle emission category; QCL total cattle stock is therefore not used for cattle allocation. In the Tier 1 diagnostic, *N* denotes the GLE stock of the emission category and is distinct from QCL total stock *TS*. The Tier 1 diagnostic characterises what the reported indicator measures, the exact Shapley decomposition attributes temporal changes, and the influence and sensitivity analyses evaluate the robustness of that attribution. The icons are schematic: in the milk-allocated panel all five species are charged, each with its own *δ* (cattle: *δ* = 1); the grey silhouettes stand for the part of the buffalo, goat, sheep and camel herds that is not milked and is removed from the numerator, not for excluded species. In the whole-herd panel the cow stands for the dairy herd, which is the only cattle herd reported. The illustration was generated with an AI image model (Section 2.7) and displays no data.

The accounting architecture is summarised in Figure 1. For country *c*, species *s* and year *t* the whole-herd intensity charges all reported methane of the herd of the species (the dairy herd for cattle, the whole national herd for the other four species) to its milk (*E* in kt CH_4_, *M* in t, so that the factor 10^6^ returns g CH_4_ kg^−1^ milk):

(1) $$I_{c,s,t}^{\mathrm{whole}}=10^{6}\;\frac{E_{c,s,t}^{\mathrm{whole}}}{M_{c,s,t}}$$

FAOSTAT scales non-bovine emissions in its Emissions intensities domain by the fraction of animals that are involved in milk production [13]. We reproduce this step with the same counts, as the ratio of animals producing milk to total stock:

(2) $$\delta_{c,s,t}=\frac{\mathrm{PA}S_{c,s,t}}{TS_{c,s,t}},\;\;I_{c,s,t}^{\mathrm{milk}}=\delta_{c,s,t}\;I_{c,s,t}^{\mathrm{whole}}$$

We chose the share of animals in milk, and not a biophysical or economic rule, for three reasons. It is built from reported counts that exist for all countries and years of the panel; it is the factor used by FAOSTAT for the same purpose [13]; and the alternatives require data that are not available globally, such as live weight sold per unit of milk for the biophysical factor of the International Dairy Federation [14,39] or prices for economic allocation. The biophysical factor is, moreover, calibrated on specialised dairy cattle and degrades at the large meat-to-milk ratios of dual-purpose herds [18,19].

Milk animals and total stock are both published FAOSTAT activity-data counts (official, estimated or imputed), rather than outputs of the allocation model itself, and the factor acts on the numerator. For cattle, the GLE category ‘Cattle, dairy’ is the milk-cow category and is associated with the milk animals of the QCL domain, whereas replacement heifers and calves are allocated to ‘Cattle, non-dairy’ [13]; the cattle numerator is therefore already restricted to milk cows and we set *δ* = 1. The two counts agree within ±5% in 79% of countries (92% of cattle milk) in 2023 and differ elsewhere; a sensitivity analysis that also scales cattle by min(1, *PAS*/GLE dairy stock) is reported in Section 3.5. The asymmetry between cattle (milk cows only) and the four other species (males, young stock and animals kept for other outputs included) is accordingly larger than the species labels suggest and is removed by Equation (2) only to the extent that *PAS*/*TS* represents herd function.

PAS/TS is a physical herd-function proxy that removes the Tier 1 contribution of non-milked animals from the milk numerator. It is not a complete co-product allocation, because emissions from milked animals are not partitioned between milk and their other outputs. The whole-herd intensity is retained as a boundary sensitivity, not as the biological comparison. The direction of the bias that remains is discussed in Section 4.2. The national intensity under either boundary is the production-weighted mixture

(3) $$I_{c,t}=\sum_{s}w_{c,s,t}\;I_{c,s,t},\;\;w_{c,s,t}=\frac{M_{c,s,t}}{\sum_{s^{\prime }}M_{c,s^{\prime },t}}$$

where the weights are shares of national milk, not herd numbers. Under Tier 1, methane equals the number of animals *N* of the emission category (the GLE stock) times a fixed emission factor, so the intensity is, to first order, the inverse of reported milk per head:

(4) $$I_{c,s,t}^{\mathrm{whole}}=\frac{EF_{c,s}\;N_{c,s,t}}{M_{c,s,t}}\;\;\Rightarrow \;\;\ln I_{c,s,t}^{\mathrm{whole}}=\ln EF_{c,s}+\ln\frac{N_{c,s,t}}{M_{c,s,t}}$$

With *EF* in kg CH_4_ head^−1^ year^−1^ and *M* in t, the right-hand side is again in g CH_4_ kg^−1^ milk. Combining Equations (2) and (4), the milk-allocated intensity reduces to the emission factor divided by the milk yield per animal in milk, *y* = *M*/*PAS* (t head^−1^ year^−1^), whenever *N* = *TS*, that is, whenever the GLE and QCL stocks coincide:

(5) $$I_{c,s,t}^{\mathrm{milk}}\approx \frac{EF_{c,s}\;\mathrm{PA}S_{c,s,t}}{M_{c,s,t}}=\frac{EF_{c,s}}{y_{c,s,t}}$$

We therefore regressed ln *I* on ln(*N*/*M*) separately by species and boundary (for the milk-allocated indicator, ln(*PAS*/*M*) is the relevant predictor), with country-clustered bootstrap intervals for the slope (500 resamples) [40]. The regression is a diagnostic of what the indicator measures; it is not an inferential model of emissions.

### 2.3. Analytical Panel and Data-Quality Audit

A country–species series enters the panel only if the required variables are positive in each of the four years: milk production, methane, milk animals and total stock for buffalo, goats, sheep and camels, and milk production and dairy-cattle methane for cattle, for which *δ* = 1 and the milk-animal and stock counts are not needed. This complete-case rule keeps the set of species of each country constant, so that changes in composition are changes in shares and not in coverage. Series that failed the rule are listed with the reason in Table S1. The retained cells carry 99.91% of the 2023 raw milk reported in the five milk items. Two accounting identities (species shares sum to one; the mixture identity of Equation (3)) were verified for each country–year and boundary.

Influential observations were examined in three ways, fixed here as analysis rules and not as pre-registered hypotheses: (i) leave-one-country-out recomputation of the global decomposition [33]; (ii) removal of the *k* = 5 and *k* = 10 countries with the largest absolute contribution to the global structural component; and (iii) removal of the cell series flagged by a denominator-collapse rule (intensity above the 99th percentile of its species and milk below one quarter of the series’ own 2020–2023 maximum).

For flagged cells we re-inspected the FAOSTAT flag, note and companion series (milk animals, stock, methane and the milk of the other species of the same country). A series was classed as an error only if it could be demonstrated as one. The series flagged by rule (iii) is excluded from the primary estimates because it fails a plausibility check (reported milk per animal in milk of a small fraction of its own value in the other years), not because an error was demonstrated, and the full panel is reported alongside as a sensitivity analysis. The denominator-collapse rule and the plausibility check were formulated after the Mongolian series had been identified by influence diagnostics of an earlier analysis and are therefore post hoc.

### 2.4. Exact Decomposition of Changes in Intensity

For each country the change in national intensity between the base year 0 (2020) and the end year 1 (2023) was split into a structural term (change in the shares of national milk supplied by each species) and a within-species term (change in species intensities) with the exact two-factor Shapley value [29,30,31,32]:

(6) $$\Delta_{c}^{\mathrm{struct}}=1/2\sum_{s}\left(w_{s}^{1}-w_{s}^{0}\right)\left(I_{s}^{0}+I_{s}^{1}\right),\;\;\Delta_{c}^{\mathrm{within}}=1/2\sum_{s}\left(w_{s}^{0}+w_{s}^{1}\right)\left(I_{s}^{1}-I_{s}^{0}\right)$$

so that Δ^struct^ + Δ^within^ = *I*^1^ − *I*^0^ holds exactly. Country terms were averaged with weights equal to national milk output averaged over the two endpoints. This global quantity is the production-weighted mean of national changes at fixed country weights. The change in the world aggregate ratio *R* additionally depends on the redistribution of milk between countries, and was decomposed exactly into three factors, the country share of world milk *p_c_*, the within-country species share *w_c_*_,*s*_ and the intensity *I_c_*_,*s*_, with the Shapley value over all orderings of the factors:

(7) $$R=\sum_{c}p_{c}\sum_{s}w_{c,s}I_{c,s},\;\;\phi_{k}=\frac{1}{3!}\sum_{\pi }\left[R(S_{\pi }^{k}\cup \{k\})-R(S_{\pi }^{k})\right]$$

The Shapley value was preferred to a logarithmic mean Divisia index because it is exact without a residual and remains defined when a species share is zero in one of the years, a case that is frequent in a panel in which most countries milk one or two species [29,30]. The decomposition does not assume that the factors are independent; it distributes the interaction term symmetrically [41].

The two estimands are different quantities and are always labelled. Both decompositions were computed under the whole-herd and the milk-allocated boundary, for the full panel and for each influence case.

### 2.5. Functional-Unit Sensitivity

To address milk composition, intensities were re-expressed per kg of fat- and protein-corrected milk, *I*^FPCM^ = *I*/*f_s_*, with *f_s_* = 0.1226 *F_s_* + 0.0776 *P_s_* + 0.2534 [14], where *F_s_* and *P_s_* are representative fat and protein percentages (cattle 4.0 and 3.3; buffalo 7.5 and 4.3; goats 3.8 and 3.4; sheep 7.0 and 5.6; camels 3.5 and 3.1) taken from the literature on species-specific correction [15,16,42,43]. The equation is calibrated on bovine milk. Its use for the other species is an approximation, and the result is reported as a composition-adjusted sensitivity analysis, not as a species-specific product equivalence. A product-yield basis such as cheese cannot be constructed from FAOSTAT country-level data.

### 2.6. Bounded Compositional Counterfactual

To show how far the reported national figure could respond to composition, each country with at least two species was given a bounded transfer of milk output, taken from its highest-intensity species and added to its lowest-intensity species, up to a budget *b* of the national 2023 milk (0.01, 0.05, 0.10 and 0.20; 0.10 as the reference) and never beyond the share held by the donor species. The problem minimises Σ*_s_ w*′*_s_ I_s_* subject to ½‖*w*′ − *w*‖_1_ ≤ *b* and no introduction of absent species. Its solution is closed-form, so the intensity change of a country equals

(8) $$\Delta I_{c}=\sum_{s\in D_{c}}m_{s}\left(I_{s}-I_{\mathrm{low},c}\right),\;\;\sum_{s\in D_{c}}m_{s}=\min\;\left(b,\sum_{s\in D_{c}}w_{s}\right)$$

where *D_c_* is the set of species with higher intensity than the lowest one and the transfers *m_s_* are taken in descending order of intensity. As an illustration, Greece in 2023 has milk-allocated intensities of 19.5 (cattle), 29.9 (goats) and 48.9 (sheep) g CH_4_ kg^−1^ and milk shares of 0.34, 0.18 and 0.48; a transfer of 0.10 from sheep to cattle changes the computed national intensity from 35.5 to 32.5 g CH_4_ kg^−1^ (−8.3%) without exhausting the represented sheep-milk share. The closed form was checked against a general-purpose linear-programming solver (HiGHS, as implemented in SciPy 1.17.1) [44] in every country and boundary (maximum difference of the objective 5 × 10^−13^ g CH_4_ kg^−1^). The quantity is an accounting counterfactual on observed intensities, with no feedback of the transfer on the intensities and no representation of feed, land, markets, product standards or cultural roles. It is expressed only as a percentage change of the reported metric; no absolute methane quantity is derived, and the figures are not estimates of achievable emission reductions. The headline summary is the production-weighted counterfactual decrease in the reported aggregate ratio; the median and interquartile range among the eligible multispecies countries are reported alongside.

### 2.7. Reproducibility and Use of Generative AI

All calculations were performed in Python 3.11.16 (Python Software Foundation, Wilmington, DE, USA) with pandas 2.3.3, NumPy 2.4.6 and SciPy 1.17.1 on the analytical panel described above, which is provided with every FAOSTAT flag and note, together with a data dictionary, as Supplementary File S1. A hierarchical Bayesian smoother of species intensities and a mean–conditional value-at-risk (CVaR) optimisation, used in the preprint version of this analysis, are not part of the analysis presented here; they are described and re-assessed for audit in the Supplementary Materials (Sections S6 and S7; Table S12). Generative AI tools were used for language editing and for software-development assistance between January and October 2026. Figure 1 and the graphical abstract are schematic illustrations generated with an AI image model (gpt-image-2.5-flare-2026-09-08; OpenAI, San Francisco, CA, USA); they contain no data, and their labels and symbols were checked by the author against Section 2.1, Section 2.2, Section 2.3, Section 2.4, Section 2.5 and Section 2.6. All other figures, including Figure S1, were not generated with AI: they were plotted in Python 3.11.16 (Matplotlib 3.11.1, with GeoPandas 1.2.0 for the map) directly from the result tables of the analysis. All outputs were reviewed and verified by the author, no tool created or altered source data or determined any methodological choice, and the author takes full responsibility for the content.

## 3. Results

### 3.1. Analytical Panel and Coverage

The analytical panel contains 405 country–species series in 186 countries (1620 cells), and carries 970.2 of the 971.1 Mt of raw milk reported in 2023. In 2023 cattle milk was reported by 185 countries, goat milk by 103, sheep milk by 71, camel milk by 29 and buffalo milk by 17; 79 countries milk a single species, and 107 milk two or more (Table S1 lists excluded series). The excluded series are small: they carry 0.89 Mt of 2023 milk, mostly buffalo milk of Venezuela (0.43 Mt) and Italy (0.25 Mt), for which the inventory has no buffalo methane or the QCL no milk-animal count, and the goat and sheep milk of Cyprus. All 2976 accounting checks (744 country–years × 2 boundaries × 2 identities) passed, with a maximum deviation of 5 × 10^−13^ g CH_4_ kg^−1^ in the mixture identity. These checks verify identities among derived quantities, not the FAOSTAT values themselves.

Of the methane charged to milk in 2023, non-cattle species carry 52% under the whole-herd boundary but 25% under milk allocation, while supplying 19% of the milk. The world aggregate ratio of the panel was 46.3 g CH_4_ kg^−1^ (whole-herd) or 29.7 (milk-allocated) in 2023, and it declined in each year of the period under both boundaries (Table S5). Of the non-bovine milk-animal counts used in the allocation, 56% are imputed values, which is considered in the limitations.

### 3.2. Effect of the Allocation Boundary on Species Intensities

**Figure 2:**
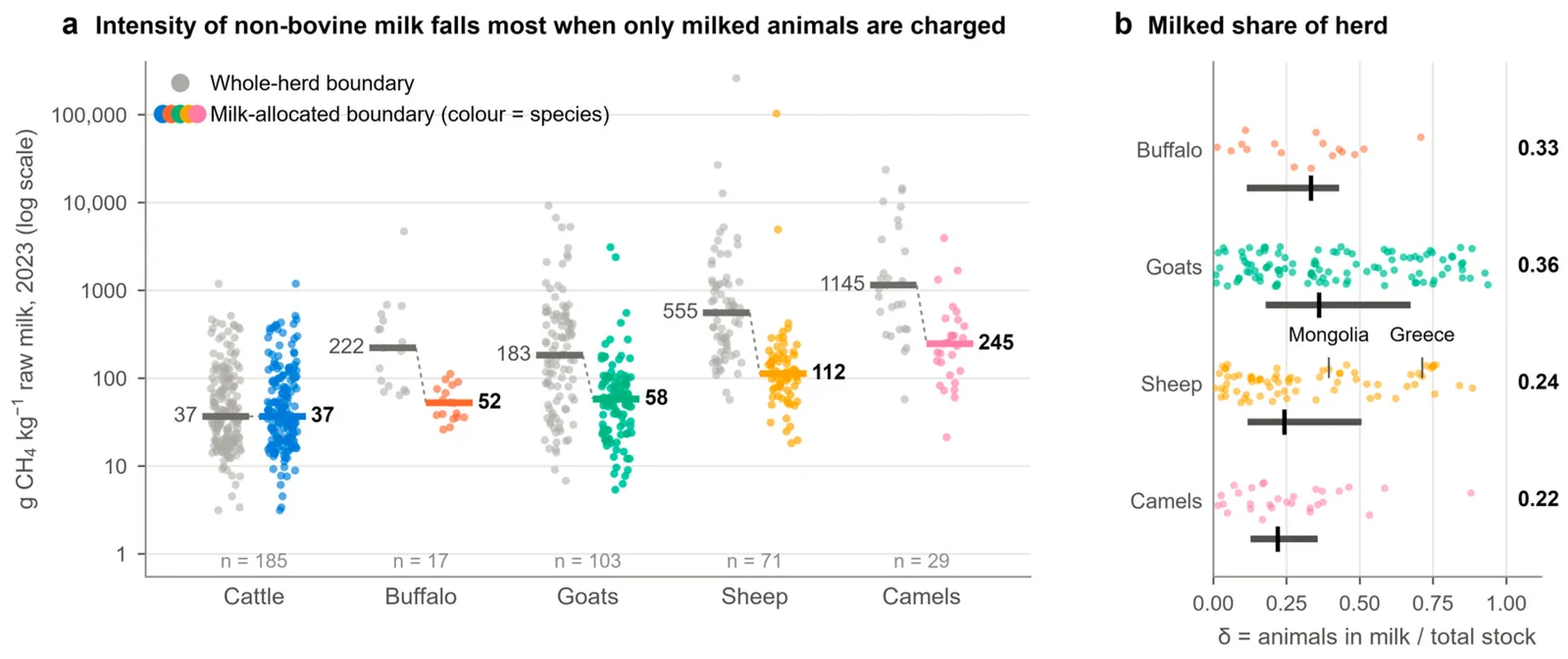
Effect of the allocation boundary on species methane intensity, 2023. (**a**) Country-level intensity (g CH_4_ kg^−1^ raw milk, logarithmic scale) under the whole-herd boundary (grey) and the milk-allocated boundary (coloured); points are countries and the thick dash is the median (value printed beside it). (**b**) Distribution across countries of the milk-allocation factor *δ* = *PAS*/*TS* for the four non-bovine species, with the interquartile range as a bar and the median as a vertical tick; cattle *δ* = 1 by construction.

Milk-animal shares are well below one for every non-bovine species (2023 medians of *δ*: buffalo 0.33, goats 0.36, sheep 0.24, camels 0.22; Figure 2b) and vary widely among countries, so no single allocation ratio per species can represent them. Under the whole-herd boundary the pooled 2023 intensity (total methane over total milk) is 27.4 g CH_4_ kg^−1^ for cattle, and 2.9, 8.5, 17.3 and 17.9 times that for buffalo, goats, sheep and camels (Table 2). With milk allocation the multiples fall to 1.08, 1.88, 5.19 and 3.89. Medians across countries show the same compression (Figure 2a): the ratio of the median goat intensity to the median cattle intensity falls from 5.0 to 1.6.

Under Tier 1, the milk-allocated intensity is the emission factor divided by milk per animal in milk (Equation (5)), so that species differences can be read from these two reported quantities. In 2023 the median emission factor per head was 73 kg CH_4_ head^−1^ year^−1^ for dairy cows, 59 for buffalo, 48 for camels and about 5 for goats and sheep, whereas the median milk per animal in milk was 1.5 t for cattle, 1.1 t for buffalo, 0.21 t for camels, 0.09 t for goats and 0.05 t for sheep. A goat has about one fourteenth of the emission factor of a dairy cow and about one seventeenth of its milk yield, which places it close to cattle once only the milked animals are charged; a camel has two thirds of the factor and one seventh of the yield. The pooled milk-allocated intensity changed little between 2020 and 2023 for every species (cattle −2.0%, buffalo −1.5%, goats −7.0%, sheep +3.0%, camels −0.7%).

Country orderings change more than the pooled ratios. Among the 107 countries that milk at least two species, cattle have the lowest intensity in 89 under the whole-herd boundary but in 53 under milk allocation, where goats are lowest in 44, buffalo in 5, camels in 3 and sheep in 2. The pattern follows herd function. In Greece, where sheep and goats are dairy flocks, *δ* is 0.72–0.77 and the observed sheep-to-cattle intensity ratio is about 2.5 under milk allocation, while in Mongolia, where flocks are kept mainly for meat and fibre, *δ* is 0.31–0.41 for the same species.

### 3.3. What the Indicator Measures: Tier 1 Structure and Panel Composition

**Figure 3:**
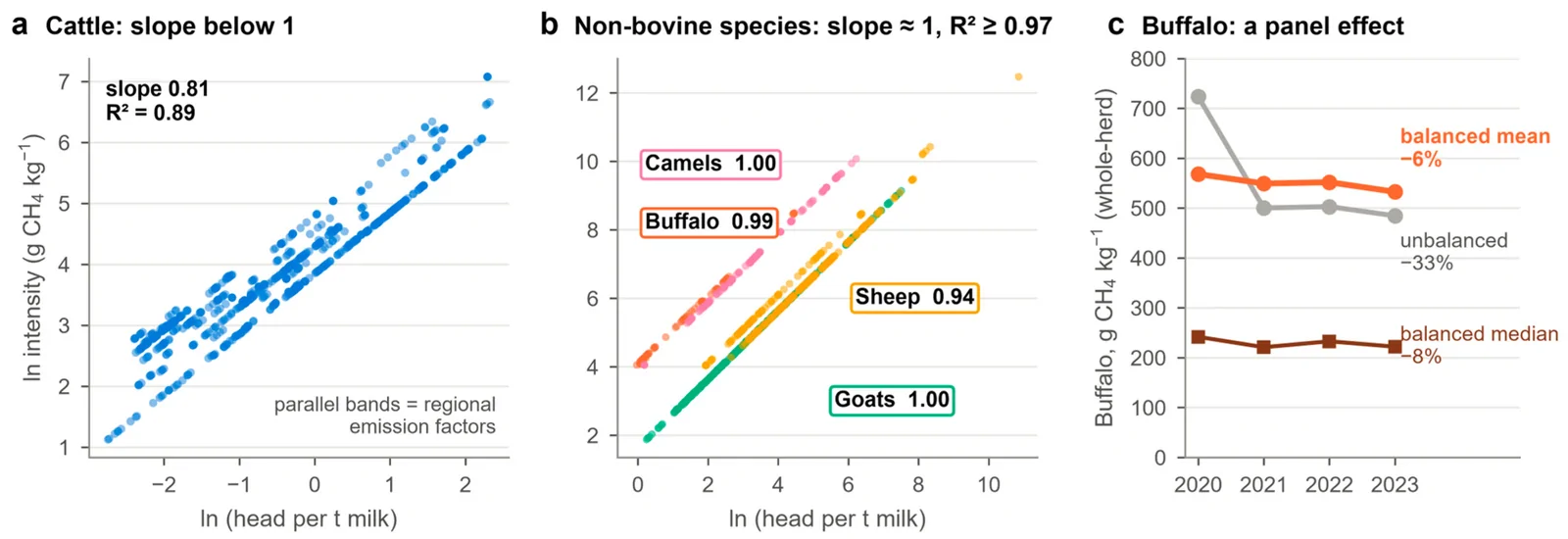
What the Tier 1 indicator measures. (**a**) Cattle (dairy herd): ln of whole-herd intensity against ln of head per tonne of milk. (**b**) The same relation for the four non-bovine species; the parallel lines reflect the fixed per-head emission factor of each species and region. (**c**) Buffalo mean intensity on the unbalanced panel and on the balanced panel of the 17 countries with complete series, and the balanced median.

For the four non-bovine species ln *I* is almost exactly a linear function of ln(*N*/*M*) (Figure 3a,b; Table S2): the whole-herd slope is 0.99 for buffalo, 1.00 for goats, 0.94 for sheep and 1.00 for camels (0.91–1.00 under the milk-allocated boundary), with R^2^ between 0.977 and 1.000 (0.960 and 0.998 under the milk-allocated boundary). The implied emission factor, *E/N*, is nearly constant within a species (median 5.17 kg CH_4_ head^−1^ year^−1^ for goats, 48 for camels, 59 for buffalo) and does not change within a country series (median coefficient of variation 0.0001%). A slope close to 1 is expected by construction, because Tier 1 methane is a fixed factor times head count; the regression checks this identity and is not an independent result. For cattle the slope is 0.81 (R^2^ = 0.89), because regional factors for dairy cows vary between about 47 and 110 kg CH_4_ head^−1^ year^−1^ (interquartile range). The within-species intensity of the four non-bovine species, and with it the within-species term of the decomposition, therefore measures changes in reported stock per tonne of milk, not changes in emissions per animal or per unit of feed.

The same structure makes ratio-based means sensitive to panel composition, as the buffalo series shows. On the unbalanced extraction the buffalo mean falls from 723 to 484 g CH_4_ kg^−1^ in three years (−33%). On the balanced panel of 17 countries the mean moves from 568 to 532 (−6.3%), the median from 242 to 222 (−8.2%) and the pooled ratio from 83.7 to 79.3. The apparent collapse is thus a property of panel composition, driven by cells that exist in only some years (for example Greek buffalo milk, 90 t in 2020, which gives 4733 g CH_4_ kg^−1^), and not a transformation of buffalo production.

### 3.4. Geographic Patterns and Low-End Values

**Figure 4:**
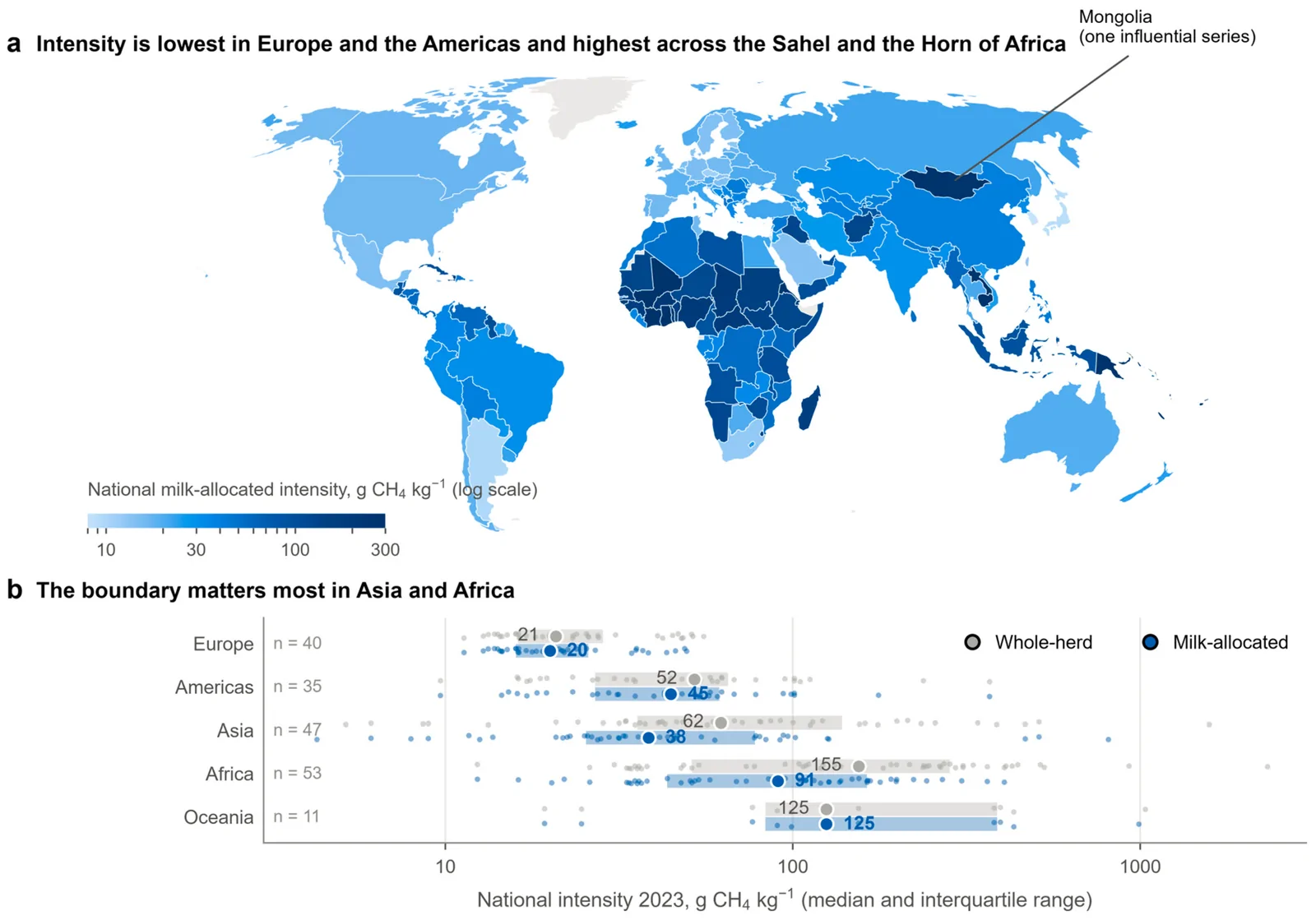
Geographic heterogeneity of national methane intensity in 2023. (**a**) Milk-allocated intensity (g CH_4_ kg^−1^ raw milk, logarithmic colour scale); values above 300 are shown in the darkest colour; grey, no data in the analytical panel. Small island states are not drawn. (**b**) National intensity by continent under the whole-herd (grey) and milk-allocated (blue) boundary; bars show the interquartile range and the circle the median. The map shows the complete 2023 analytical panel; the Mongolian sheep-milk series is screened only in the primary decomposition analysis. Base map: Natural Earth (public domain).

Pooled over all milk, milk-allocated intensity is lowest in Europe (18.9 g CH_4_ kg^−1^) and the Americas (21.7), higher in Asia (32.6) and highest in Africa (87.5) (Figure 4; Table 3; all countries in Table S3). The median of national values gives a different order (Europe 20.0, Asia 38.4, Americas 44.5, Africa 90.6) because the Americas are dominated by a few very large, efficient producers beside many small, low-yield systems; the median of Oceania (125.0) reflects small island states. Non-bovine species supply 37.0% of milk in Asia and 20.4% in Africa, against 2.5% in Europe and 0.5% in the Americas, so the boundary matters most in the first two regions: the pooled intensity of Asia falls from 58.1 to 32.6 g CH_4_ kg^−1^ with milk allocation and that of Africa from 161.3 to 87.5, whereas that of Europe changes by 1.7 and that of the Americas by 0.9 g CH_4_ kg^−1^. The two boundaries coincide in Oceania because non-bovine milk is negligible there.

The low national values queried for Israel (5.2 g CH_4_ kg^−1^ whole-herd, 4.3 milk-allocated), Cyprus (6.1) and the Republic of Korea (8.6) follow from the Tier 1 identity. The dairy herds of these countries have reported yields of 14.2 t (Israel), 7.9 t (Cyprus) and 10.0 t (Korea) per milk cow and year, against Tier 1 factors of 48, 48 and 78 kg CH_4_ head^−1^ year^−1^, which gives 3.4, 6.1 and 7.8 g CH_4_ kg^−1^ for cattle. Cattle methane is present for all three; goat and sheep methane is present for Israel in all years and goat methane for Korea, whereas GLE publishes no goat or sheep methane for Cyprus in 2023 although QCL reports 37,280 t of goat and 49,620 t of sheep milk. The Cyprus small-ruminant series are therefore excluded by the complete-case rule (Table S1), so the Cyprus value is cattle-only in all years; it is a coverage gap in the source, not a low estimate for the species. The low values of Israel and the Republic of Korea are consequences of fixed regional Tier 1 factors applied to very high reported yields, not evidence of exceptionally low emissions; the three countries are marked in Table S3.

### 3.5. Decomposition of the Change in Intensity and Its Sensitivity to One Influential Series

**Figure 5:**
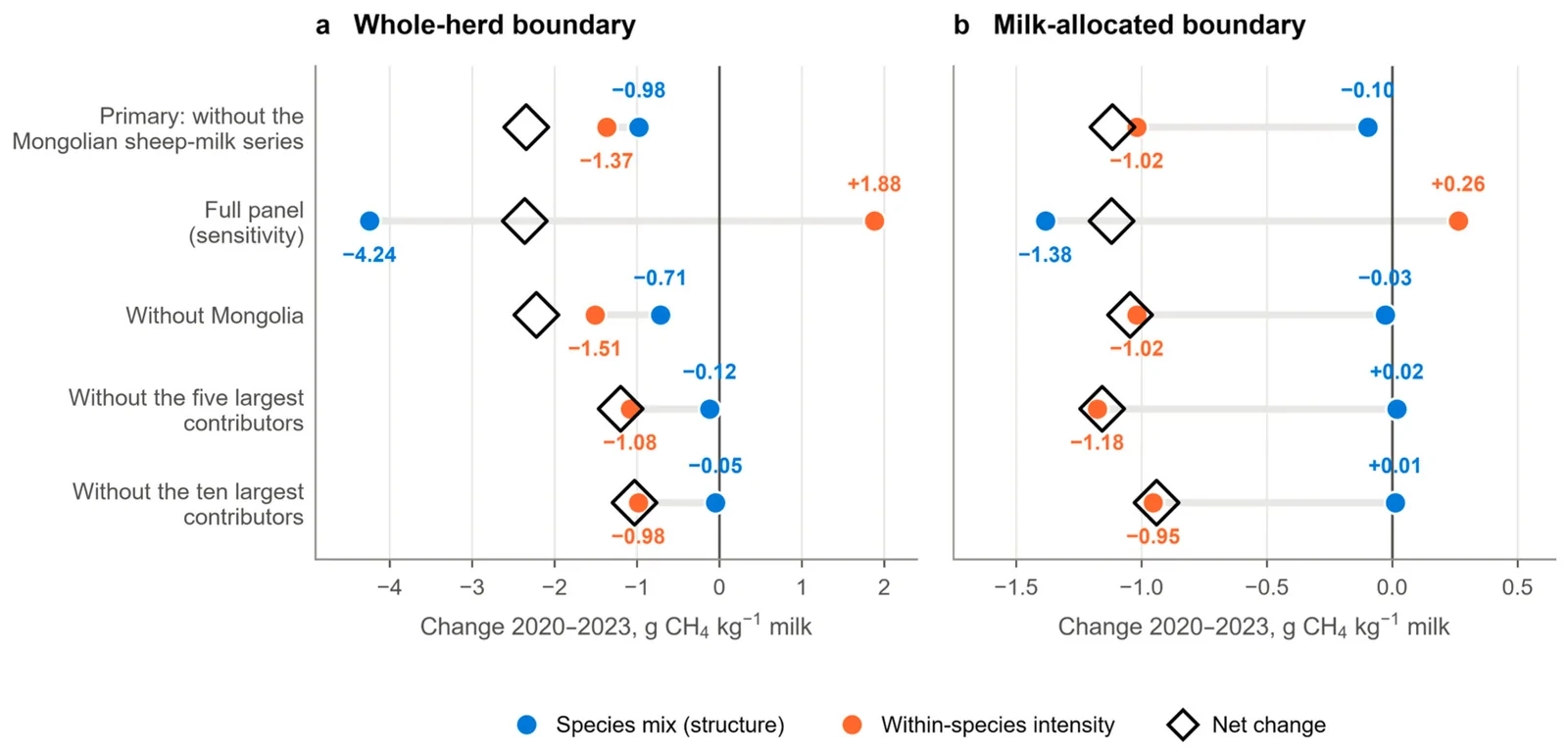
Robustness of the global decomposition to influential records. Two-factor Shapley components (g CH_4_ kg^−1^ raw milk, production-weighted mean of national changes, 2020–2023) under (**a**) the whole-herd boundary and (**b**) the milk-allocated boundary for the primary case (Mongolian sheep-milk series excluded), the full panel (sensitivity), without Mongolia, without the five largest structural contributors and without the ten largest. Blue circles show the species-mix (structural) component, orange circles the within-species intensity component and open diamonds the net change.

The world aggregate ratio fell from 47.38 to 46.30 g CH_4_ kg^−1^ under the whole-herd boundary (−1.08) and from 30.38 to 29.74 under milk allocation (−0.64; also −0.64 without the Mongolian sheep-milk series). The change is small and, under milk allocation, stable: it is −0.64 on the full panel and −0.67 without the five largest contributors, whereas under the whole-herd boundary the same exclusion moves it from −1.08 to −0.29. Its attribution is not stable (Table 4; Figure 5). Two different decompositions are reported and are not alternative estimates of one quantity (Section 2.4).

In the primary case, in which the Mongolian sheep-milk series is screened (Section 2.3), the three-factor decomposition of the world ratio under milk allocation gives country shares of +0.48 g CH_4_ kg^−1^, species mix −0.10 and country–species intensity −1.02 (sum −0.64); with the series retained the values are +0.57, −1.43 and +0.21 (sum −0.64). In the fixed-country-weight two-factor decomposition (the production-weighted mean of national changes) the full-panel structural term is −4.24 g CH_4_ kg^−1^ under the whole-herd boundary and −1.38 under milk allocation, with within-species terms of +1.88 and +0.26.

A single country–species series explains most of it: removing Mongolian sheep milk reduces the structural term to −0.98 (whole-herd; 77% removed) and −0.10 (milk-allocated; 93% removed) and turns the within-species term negative (−1.37 and −1.02). Excluding all of Mongolia gives −0.71 and −0.03; excluding the five largest contributors gives −0.12 and +0.02, and ten gives −0.05 and +0.01. In the leave-one-country-out analysis the structural term stays between −1.84 and −1.38 (milk-allocated) when any country other than Mongolia is removed, but is −0.03 when Mongolia is removed (Table S8). In 2020–2023 the denominator-collapse rule flags this series and no other.

The cell is not an obvious transcription error. FAOSTAT carries Mongolian sheep milk at 21,617 t in 2022 and 572.5 t in 2023 (−97%), flagged as an official figure, while sheep methane moves from 167.0 to 150.0 kt and the reported milk-animal count from 12.97 to 11.61 million head. The intensity therefore rises from 7726 to 262,006 g CH_4_ kg^−1^ (whole-herd). In the same year goat milk falls by 69%, camel milk by 34% and cattle milk rises by 69%, while total reported Mongolian milk moves from 221 to 265 kt. With 11.6 million animals in milk, the 2023 value corresponds to 49 g of milk per animal in milk and year, 3% of the 1.6–1.7 kg of 2020–2022. The screening criterion is this relative collapse within the series, with stable animal counts, and not the absolute level: even 1.6–1.7 kg is more than an order of magnitude below the median of about 50 kg for sheep (Section 3.2), which suggests that the Mongolian milk-animal count covers breeding females in general and not only animals actually milked.

A change in the species attribution of milk in the national series would be consistent with this pattern, and we checked it against the national statistics. The National Statistics Office of Mongolia reports total milk of 220,739 t in 2022 and 266,254 t in 2023 (+20.6%) [46]. The sum of the five FAOSTAT milk items of Mongolia equals this national total to within 0.1 t in each of 2020–2022 and is 0.6% below it in 2023, and the FAOSTAT stocks of sheep, goats and camels equal the national livestock census counts (details in Section S5 of the Supplementary Materials) [46].

The national total and the livestock numbers are therefore consistent with the national statistics, and what changes abruptly in 2023 is the split of milk among species: reported milk per animal in milk rises for cattle and falls for goats and sheep (Table S9). The public tables give no species split, so this allocation could not be reconciled at source. The series is therefore treated as an implausible, externally unverified reporting discontinuity and is screened from the primary estimates (Section 2.3); the full panel is reported as a sensitivity analysis.

When a denominator of one cell collapses, the change in the species share and the change in the species intensity are the same event, and the Shapley split assigns it to two terms of opposite sign and similar size (Mongolia, milk-allocated: structure −5833 and within-species +5515 g CH_4_ kg^−1^; whole-herd: −15,191 and +14,581); this is a property of the decomposition applied to a collapsing cell and not a finding.

Two further results follow. First, the geographical term of the world-ratio decomposition is stable (+0.57 with and +0.48 without the Mongolian series, milk-allocated), because world milk shifted towards countries with above-average intensity. Second, when the influential series is removed and milk is allocated, species mix contributes almost nothing (−0.10 to the world ratio and −0.10 to the fixed-weight mean) and the decline is carried by within-species terms (−1.02), which, as shown in Section 3.3, are changes in reported stock per tonne of milk. The functional unit changes the levels (the world ratio in 2023 is 27.5 per kg of fat- and protein-corrected milk against 29.7 per kg of raw milk, both in grams of methane) but not this conclusion: per kg FPCM the structural term is −1.16 on the full panel and −0.02 without the Mongolian series. Using the GLE stock instead of the QCL stock as total stock in *δ* gives −1.38 and +0.31, essentially the same. Scaling cattle also by milk animals over the GLE dairy stock (capped at one) gives −1.38 and +0.28 on the full panel and −0.10 and −1.01 without the Mongolian series (all cases in Table S7).

Over longer windows (2015–2023 and 2010–2023), the screened primary analysis gives decreases in the world ratio of 3.64 and 6.54 g CH_4_ kg^−1^ under milk allocation. The decline is carried by the within-species term (−4.56 and −8.65 g CH_4_ kg^−1^), whereas the species-mix term remains small (−0.03 and −0.06 g CH_4_ kg^−1^); the attribution is therefore not specific to the four-year window (Table S11). In the 2010–2023 window the denominator-collapse rule also flags the Russian sheep-milk series, which is then screened together with the Mongolian one.

At country level, the structural term is negative in 61 countries, positive in 45 and zero in 80: the 79 single-species countries and Bangladesh, where the four species shares are identical in all four years (0.900, 0.070, 0.025 and 0.005 for cattle, goats, buffalo and sheep, all values being FAOSTAT estimates, which suggests imputation). The within-species term is negative in 112, positive in 73 and zero in 1 (milk-allocated; the whole-herd boundary gives the same counts for the structural term). The largest negative contributions to the production-weighted within-species term, excluding Mongolia, come from India (−0.31 g CH_4_ kg^−1^), Bangladesh (−0.16) and Nepal (−0.09); in all three, reported milk grew faster than reported stock between 2020 and 2023 (cattle milk +17% against +10% stock in India, +32% against +2% in Bangladesh and +32% against −21% in Nepal), which under Tier 1 lowers the intensity mechanically (Section 3.3). The full country-level decomposition is given in Table S4.

### 3.6. Sensitivity of Reported Intensity to a Bounded Compositional Counterfactual

At a compositional-transfer budget of 10% of national milk, the production-weighted counterfactual decrease in the reported aggregate intensity is 7.2% under milk allocation and 23.4% under whole-herd accounting (Table 5; Figure S1; all budgets in Table S10). These percentages quantify the arithmetic sensitivity of the reported intensity metric under fixed observed species-specific intensities; they are not estimates of achievable methane-emission reductions. Among the 107 eligible countries the median counterfactual decrease is 7.3% (interquartile range 4.1–14.2) under milk allocation and 17.5% (7.4–32.4) under whole-herd accounting. The direction of the transfer also depends on the boundary: under whole-herd allocation it points to cattle in 89 countries and to goats or camels in the other 18, whereas under milk allocation it points to cattle in 53 countries, to goats in 44, to buffalo in 5, to camels in 3 and to sheep in 2. The five largest contributions account for 53% of the production-weighted figure under milk allocation.

A budget of 10% is large relative to the minority sectors: the transfer exhausts the represented milk shares of all higher-intensity donor species in 33 of 107 countries under milk allocation (42 under the whole-herd boundary; for example, Türkiye, Italy, Iran and Saudi Arabia, where the donor species supply less than 10% of national milk), so that in these countries the computed figure is bounded by the mathematical exhaustion of the represented sheep, goat or buffalo milk shares and not by the budget. The result is insensitive to the Mongolian series (production-weighted counterfactual decrease 7.0%) and to the functional unit (7.0% per kg FPCM).

## 4. Discussion

### 4.1. Main Findings

The comparison of ruminant species by methane per kilogram of milk depends on how herd emissions are allocated to milk. Charging whole-herd methane to milk makes buffalo, goat, sheep and camel milk 2.9 to 17.9 times as intense as cattle milk; allocating methane with the share of animals that are milked reduces this range to 1.1–5.2 times, and cattle are the lowest-intensity species in 53 of 107 multispecies countries, compared with 89 under whole-herd allocation. Three further results qualify any biological reading. The within-species intensity of the non-bovine species is essentially the inverse of reported stock per tonne of milk, as expected from Tier 1 accounting. The aggregate change in the world ratio is small and, under milk allocation, stable. And its split between species composition and within-species change depends on one implausible, externally unverified national series. These are the findings that the evidence supports; they are statements about an accounting system and not rankings of animals.

On the whole-herd boundary and the full panel the structural and within-species terms (−4.24 and +1.88 g CH_4_ kg^−1^) are close to those obtained on the 181-country panel of the preprint version of this analysis (−4.28 and +1.85) [47], as expected from nearly identical inputs; the five recovered entities and the complete-case rule change them by less than 0.05. The same terms shrink by a factor of three under milk allocation, and by a factor of four to more than ten when one series is removed.

### 4.2. What Cross-Species Intensity Comparisons Can and Cannot Show

A Tier 1 inventory does not observe feed, yield or herd management. It multiplies reported head counts by factors that depend only on region and category. For goats, sheep, camels and buffalo the log–log relation of Section 3.3 is nearly exact (whole-herd slopes 0.94–1.00; 0.91–1.00 milk-allocated), and the factors are constant within series, so differences in intensity between countries and years are differences in reported stock per tonne of milk. This does not make the indicator meaningless: stock per unit of milk is a legitimate indicator of production-system extensiveness and herd multifunctionality, which is the likely reason why the species rank as they do [21,48]. It does mean that the within-species term of a decomposition is not a measure of efficiency gains or of mitigation, and that the finding of a ‘positive’ or ‘negative’ within-species change must not be read as an emission effect. The same applies to the buffalo mean: its apparent fall by one third on an unbalanced panel disappears on the balanced panel (Section 3.3), which illustrates how panel composition can mimic a biological trend.

Equation (5) also shows why species orderings are not independent evidence about the animals. Under milk allocation the intensity ratio of two species in the same country is the ratio of their Tier 1 emission factors multiplied by the inverse ratio of their reported yields per animal in milk. A goat has an emission factor of about 5 kg CH_4_ per year against about 73 kg for a dairy cow, and a reported yield of about 0.09 t against about 1.5 t, so it falls near cattle, and in some countries below them. That goats are the lowest-intensity species in 44 of 107 multispecies countries under milk allocation (15 in Africa, 15 in Europe, 8 in the Americas, 5 in Asia and 1 in Oceania) is therefore a statement about default factors and reported yields, not evidence that goat milk is less emission-intensive in any physiological sense.

Allocating methane with the share of animals that are milked is a physical proxy for herd function. It does not value the meat, wool, hides, manure or draught power that the remaining animals supply, and it assigns replacement stock and males to non-milk outputs although they are part of the milk system. Biophysical and economic allocation rules lead to different shares, and the standard linear biophysical factor is calibrated on specialised dairy cattle and degrades at the larger meat-to-milk ratios of dual-purpose herds [14,18,20,39]. The allocation used here nevertheless has three advantages over whole-herd charging: it uses reported counts, it takes the country-specific milked fraction into account, and it is the one used by FAOSTAT itself [13]. The herd-function contrast that drives national orderings is visible in the data: Greek sheep and goats, kept as dairy flocks, have *δ* of 0.72–0.77, whereas Mongolian small ruminants have 0.31–0.41. A single ratio per species cannot capture it.

Two effects of opposite sign remain unquantified, and the net direction of the bias is unknown. Tier 1 emission factors for goats, sheep, camels and buffalo are averages over the whole animal category, whereas milked animals, with a higher feed intake, probably emit more than the herd average; scaling by *δ* then tends to understate non-bovine intensity relative to cattle, whose factor is specific to dairy cows. Not partitioning the methane of milked animals between milk and meat, fibre or draught power, on the other hand, tends to overstate it. The contrast between Greece and Mongolia should also be read with caution: the Mongolian milk-animal count seems to include breeding females that are not milked (Section 3.5), so *δ* probably overstates the milked share there; the direction of the contrast is robust, its size is not.

Three limits remain. Country studies document large gradients of cattle and buffalo intensity between production systems and agro-ecological zones [25,34], which Tier 1 factors cannot represent: they do not vary with breed, diet or climate within a region, so the data cannot show whether the animals of two countries differ physiologically, whether in the way suggested by studies of local breeds [8] or by evidence on cattle, goat and camel physiology [49,50,51,52]. Milk composition differs between species, and the FPCM correction applied here uses bovine-calibrated coefficients; species-specific energy- or solids-based equations are the natural refinement [15,16]. Finally, methane mass is only a subset of the farm-gate greenhouse-gas basket, and the ranking by methane need not be the ranking by all gases or by a different GWP metric [53,54].

Two features of the data limit what the allocation can resolve. First, the milk-animal count is an inventory figure whose definition may differ among national statistical systems; it can include animals in milk only for part of the year or exclude animals milked for subsistence, and more than half of the non-bovine counts are imputed. Second, *δ* is a ratio of two counts and is bounded above by one, so that a country whose reported milk is collapsing, as in Mongolia, keeps a stable *δ* while its intensity explodes; the allocation does not protect against collapsing denominators. Both features argue for reading country values as indicative and for relying on pooled, production-weighted summaries and on influence analysis rather than on single national figures.

### 4.3. Influential Records and Reporting Discontinuities

The finding that one country–species series carries 93% of the fixed-weight structural term under milk allocation (and Mongolia as a whole 98%) is a caution for any decomposition of aggregated livestock indicators. Exact decompositions allocate a collapsing denominator to the species-share and species-intensity terms symmetrically, so a single, influential and externally unverified data point can generate two large, opposite and meaningless components. The aggregate change moves by about 4% when Mongolia is removed, which is why the world-ratio change can be stated with confidence under milk allocation and its attribution cannot. Influence analysis therefore belongs next to the decomposition, and the full-panel and the screened results should be reported together.

Against the national statistics, the Mongolian milk total and livestock numbers are consistent, but the species split of milk could not be reconciled, and we do not call the series an error. Cyprus illustrates a second kind of discontinuity, a source that omits methane for species that are present in the milk statistics; the complete-case rule keeps such gaps from altering the species set within a series but does not remove their cost in coverage.

### 4.4. Interpretation of the Counterfactual

The bounded counterfactual should be read as a measure of sensitivity of the reported metric to composition, and not as mitigation potential or as a basis for sectoral substitution. A transfer of 10% of national milk exhausts the represented milk shares of all higher-intensity donor species in 33 of 107 countries under milk allocation, which for Türkiye means the mathematical exhaustion of the represented sheep, goat and buffalo milk shares and for Italy that of the sheep and goat milk shares, in a Mediterranean sector that includes products such as Pecorino Romano, whose product standards specify the species and the milk composition [55]. The Italian figures cover cattle, goat and sheep milk only, because the 0.25 Mt of Italian buffalo milk (and the 0.02 Mt of Romanian buffalo milk) is excluded for lack of a milk-animal count (Table S1). The direction of the transfer also changes with the boundary, so that no universal ‘move towards cattle’ can be derived.

In extensive systems the same animals provide meat, transport and drought insurance, and species substitution is constrained by rangeland, thermal adaptation, demand, processing and culture. For these reasons the production-weighted counterfactual decrease is the only summary reported in the main text, and the unweighted mean across countries, which includes 79 structural zeros and gives equal weight to very small producers, is relegated to the Supplementary Materials (Table S10). Mitigation research acts on realised emissions through nutrition, breeding and management [56,57] and is not replaced by this accounting exercise.

### 4.5. Implications for Inventories and Comparative Assessments

Four practical consequences follow for studies that compare milk species or decompose national dairy intensity. First, the allocation boundary and the functional unit should be stated with every figure; the same species ranking can be obtained or reversed by changing either. Second, panels should be balanced across years before trends are read, because entry and exit of reporting countries alone can produce changes larger than any plausible biological one (Section 3.3). Third, influence analysis (leave-one-out and screening of collapsing denominators) should accompany any exact decomposition, and the aggregate change should be reported next to its attribution.

Fourth, because Tier 1 intensity of the non-bovine species mirrors reported stock per tonne of milk, intensity changes should be read as changes in reported production systems and statistics, and emissions-based claims should be reserved for inventories that use country-specific (Tier 2) activity and feed data [9,10,11]. For inventory compilers the results point to the value of reporting species-specific milk animals and milk yields, which already exist in FAOSTAT but are imputed in a large share of cells, and of documenting reclassifications of milk between species such as the one suspected in the Mongolian series.

### 4.6. Limitations and Further Work

The main limitations are as follows. (i) The main window is four years, so trends are interval comparisons; windows beginning in 2015 and 2010 give the same qualitative attribution (Table S11). (ii) FAOSTAT milk-animal and stock counts are partly estimated or imputed (imputed values form the largest class of non-bovine milk-animal counts, see Section 2.1), so that *δ* carries an uncertainty that is not propagated here, and the QCL and GLE stocks differ in 28% of non-bovine cells; using GLE stocks does not change the decomposition materially (full-panel structural term −1.38 with either stock); the buffalo milk of Italy and Romania is excluded for lack of milk-animal counts, so their national figures omit buffalo (Table S1). (iii) Methane only, from enteric fermentation and manure management, is analysed; nitrous oxide and manure applied to soils or left on pasture, which are part of the FAOSTAT intensity basket, are outside the boundary [13].

(iv) The counterfactual assumes unchanged species intensities after the transfer. (v) The species split of Mongolian milk in 2022–2023 could not be reconciled with national species-level statistics. (vi) Country-level uncertainty of the underlying Tier 1 factors is not propagated. (vii) The intensities were not compared with the published FAOSTAT intensities, which are expressed per kilogram of fat- and protein-corrected milk in carbon-dioxide equivalents and include nitrous oxide. Further work should combine Tier 2 inventories where available, species-specific composition equations, product-yield bases and verified national series, and should test whether allocation by milk-animal share predicts the herd-function differences that it is meant to represent.

## 5. Conclusions

The main message of this study is that the methane intensity of buffalo, goat, sheep and camel milk relative to cattle milk depends more on how herd methane is allocated to milk than on the animals themselves. When non-bovine herd methane is allocated to milk with the country-specific share of animals in milk, instead of being charged entirely to milk, pooled intensity ratios to cattle fall from 2.9–17.9 to 1.1–5.2, and cattle have the lowest intensity in 53 instead of 89 of the 107 countries that milk more than one species. Between 2020 and 2023 the world methane intensity of milk decreased slightly, mainly through changes within species rather than through a shift in species mix; this attribution is reversed when a single implausible Mongolian sheep-milk record is retained. Because Tier 1 intensities of the non-bovine species largely mirror reported animals per tonne of milk, these figures describe an accounting system; they do not measure the biological efficiency of the animals and are not a basis for replacing one milk species with another. Comparisons of milk species and decompositions of national dairy intensity should therefore state the allocation boundary and the functional unit with every figure and report how single influential records affect the results.

## Figures and Tables

**Table 1:** Variables used in the analysis, with source, unit and analytical role.

Symbol	Variable	Source (FAOSTAT Element)	Unit	Role
*E* ^whole^	Methane of the herd of a species (enteric + manure management): whole national herd for buffalo, goats, sheep and camels; dairy herd only for cattle (GLE item 960, ‘Cattle, dairy’)	GLE: 72254 + 72256 (items 960, 946, 1016, 976, 1126)	kt CH_4_	Numerator
*M*	Raw milk production	QCL: 5510 (items 882, 951, 1020, 982, 1130)	t	Denominator
*PAS*	Animals producing milk	QCL: 5318 Milk animals	head	Allocation
*TS*	Total stock of the species (QCL)	QCL: 5111 Stocks (live animals)	head	Allocation
*N*	Number of animals of the emission category (Tier 1 activity data)	GLE: 5111 Stocks	head	Diagnostic
*δ*	Milk-allocation factor, *PAS*/*TS* (cattle = 1)	Derived	fraction	Allocation
*I*^whole^, *I*^milk^	Methane intensity, whole-herd and milk-allocated	Derived	g CH_4_ kg^−1^ milk	Outcome
*w*	Species share of national raw milk	Derived	fraction	Decomposition
*EF*	Implicit emission factor, *E*^whole^/*N*	Derived (Tier 1)	kg CH_4_ head^−1^ year^−1^	Diagnostic

GLE, FAOSTAT Emissions from Livestock; QCL, FAOSTAT Crops and Livestock Products. Item codes: cattle (dairy herd) 960/882, buffalo 946/951, goats 1016/1020, sheep 976/982, camels 1126/1130 (methane item/milk item).

**Table 2:** Methane intensity (g CH_4_ kg^−1^ raw milk) by species in 2023 under the whole-herd and the milk-allocated boundary.

Species	*n*	Milk (Mt)	*δ* (Median)	Whole-Herd, Pooled	Whole-Herd, Median (IQR)	Milk-Allocated, Pooled	Milk-Allocated, Median (IQR)	Pooled Ratio to Cattle (Whole/Milk)
Cattle	185	785.7	1	27.4	36.7 (20–94)	27.4	36.7 (20–94)	1.0/1.0
Buffalo	17	150.0	0.33	79.3	221.8 (81–453)	29.5	52.2 (36–75)	2.9/1.1
Goats	103	20.7	0.36	233.7	182.7 (56–507)	51.5	57.9 (26–99)	8.5/1.9
Sheep	71	9.8	0.24	474.6	554.8 (204–1194)	142.2	112.2 (76–169)	17.3/5.2
Camels	29	4.1	0.22	490.7	1145.2 (368–3803)	106.5	245.5 (121–390)	17.9/3.9

*n*, number of countries; *δ*, milk-allocation factor *PAS*/*TS*; pooled, total methane over total milk of the species; IQR, interquartile range across countries. Balanced panel of complete series (Section 2.3; number of countries per species in Table S6).

**Table 3:** National methane intensity (g CH_4_ kg^−1^ raw milk) by region in 2023.

Region	Countries	Milk (Mt)	Non-Bovine Milk (%)	Pooled Whole-Herd	Pooled Milk-Allocated	National Median Whole-Herd (IQR)	National Median Milk-Allocated (IQR)
Africa	53	53.5	20.4	161.3	87.5	155.1 (51–283)	90.6 (43–164)
Americas	35	201.8	0.5	22.6	21.7	52.1 (27–65)	44.5 (27–62)
Asia	47	450.8	37.0	58.1	32.6	62.2 (36–139)	38.4 (25–78)
Europe	40	234.4	2.5	20.6	18.9	20.8 (16–28)	20.0 (16–26)
Oceania	11	29.7	0.0	23.4	23.4	125.0 (83–388)	125.0 (83–388)

Regions follow the United Nations continent grouping [45]. Pooled, total methane over total milk of the region; IQR, interquartile range across countries. Country-level values are in Table S3.

**Table 4:** Decomposition of the 2020–2023 change in methane intensity (g CH_4_ kg^−1^ raw milk) under both boundaries and influence cases.

Case	Boundary	Structure	Within-Species	Net	Δ World Ratio	Country Shares	Species Mix	Intensity
Mongolian sheep-milk series excluded (primary)	Whole-herd	−0.98	−1.37	−2.34	−1.07	+1.29	−0.98	−1.37
	Milk-allocated	−0.10	−1.02	−1.12	−0.64	+0.48	−0.10	−1.02
Full panel (sensitivity)	Whole-herd	−4.24	+1.88	−2.36	−1.08	+1.52	−4.35	+1.75
	Milk-allocated	−1.38	+0.26	−1.12	−0.64	+0.57	−1.43	+0.21
Mongolia excluded	Whole-herd	−0.71	−1.51	−2.22	−1.03	+1.18	−0.71	−1.50
	Milk-allocated	−0.03	−1.02	−1.04	−0.62	+0.43	−0.03	−1.02
Five largest contributors excluded	Whole-herd	−0.12	−1.08	−1.20	−0.29	+0.91	−0.12	−1.08
	Milk-allocated	+0.02	−1.18	−1.16	−0.67	+0.48	+0.02	−1.17
Ten largest contributors excluded	Whole-herd	−0.05	−0.98	−1.03	−0.28	+0.75	−0.05	−0.98
	Milk-allocated	+0.01	−0.95	−0.94	−0.57	+0.37	+0.01	−0.95
FPCM functional unit	Milk-allocated	−1.16	+0.21	−0.95	−0.65	+0.37	−1.19	+0.17
FPCM, Mongolian sheep-milk series excluded	Milk-allocated	−0.02	−0.94	−0.96	−0.64	+0.32	−0.02	−0.94

Left block: exact two-factor Shapley decomposition aggregated with fixed country weights (production-weighted mean of national changes). Right block: exact three-factor decomposition of the change in the world aggregate ratio (Δ world ratio = country shares + species mix + intensity). FPCM, fat- and protein-corrected milk. Different estimands: the two blocks are not alternative estimates of one quantity. The primary case is the Mongolian sheep-milk series excluded; the full panel is a sensitivity analysis. Components may not sum exactly because of rounding.

**Table 5:** Sensitivity of the reported aggregate intensity to a bounded compositional counterfactual on observed 2023 intensities.

Boundary	Budget *b*	Production-Weighted Counterfactual Decrease in Reported Intensity (%)	Median Counterfactual Decrease in Reported Intensity, Eligible Countries (IQR) (%)	Donor Shares Exhausted (*n* of 107)
Whole-herd	1%	7.0	3.8 (1.4–9.8)	17
	5%	16.9	14.5 (5.2–23.1)	32
	10%	23.4	17.5 (7.4–32.4)	42
	20%	27.9	21.4 (10.9–43.8)	56
Milk-allocated	1%	1.5	1.3 (0.7–2.3)	12
	5%	4.6	4.7 (2.5–8.1)	25
	10%	7.2	7.3 (4.1–14.2)	33
	20%	10.0	12.7 (6.3–21.1)	40

Accounting sensitivity of a reported metric: no causal, agronomic or economic feasibility is implied, and the percentages are not estimates of methane-emission reductions. Eligible countries milk at least two species (*n* = 107); cattle have the lowest intensity in 89 (whole-herd) and 53 (milk-allocated) of them at every budget. “Donor shares exhausted” counts countries in which the transfer exhausts the represented milk shares of all higher-intensity donor species. IQR, interquartile range.

## Data Availability

The source data are publicly available from FAOSTAT, domains QCL and GLE, at https://www.fao.org/faostat/ (accessed on 2 October 2026); the items, elements and units are listed in Table 1, and national totals and livestock numbers used to check the Mongolian series are from the National Statistics Office of Mongolia at https://data.1212.mn (accessed on 2 October 2026) [46]. The analytical panel used for all results, with FAOSTAT flags and notes and a data dictionary, is provided as Supplementary File S1. The FAOSTAT extraction script (revision_R1/R1_code/01_extract_faostat.py), the analytical panel, the data dictionary, the analysis code and all result tables of the present analysis are deposited in the public GitHub repository https://github.com/ketney1982/global-milk-emissions-inequality (tag v4.0.1-R1, commit 7545379; accessed on 15 October 2026); the repository has been public since February 2026, and the commands given in its README regenerate the results reported in Table 2, Table 4 and Table 5; earlier, preprint-stage versions of the pipeline remain available under tags v3.0.1-R3 and v3.1.0-R4, which do not refer to reviewers or revisions.

## Supplementary Materials

The following supporting information can be downloaded at: https://www.mdpi.com/article/10.3390/ani16203273/s1, Document S1: Supplementary methods, tables and figures, including Table S1: Country–species series excluded from the analytical panel; Table S2: Regression of ln intensity on ln(N/M) (ln(PAS/M) for the milk-allocated boundary) by species and boundary, 2020–2023; Table S3: National methane and methane intensity in 2023 under the whole-herd and the milk-allocated boundary, all countries of the analytical panel; Table S4: Country-level exact Shapley decomposition of the 2020–2023 change in national intensity; Table S5: World aggregate ratio of the analytical panel; Table S6: Number of countries per species in the balanced analytical panel; Table S7: All influence and sensitivity cases of the decomposition; Table S8: Leave-one-country-out analysis of the production-weighted structural term; Table S9: Mongolia: national milk total and species-level FAOSTAT milk, 2020–2023; Table S10: Sensitivity of the reported intensity to a bounded compositional counterfactual: all budgets and cases; Figure S1: Sensitivity of the reported intensity to a bounded compositional counterfactual; Table S11: Sensitivity of the aggregate decomposition to the length of the study window; Table S12: Posterior reference intensity of the smoother of the preprint version against the observed national intensity for the 20 largest milk producers of the preprint panel. Sections S1–S8 of Document S1 cover the extraction specification, excluded series, Tier 1 diagnostics, country results, sensitivity analyses, the hierarchical smoother and the mean–CVaR optimisation of the preprint version, and data and reproducibility. File S1: Analytical panel with FAOSTAT flags and notes, and data dictionary (CSV). References [44,46,58] are cited in the Supplementary Materials.

## Abbreviations

The following abbreviations are used in this manuscript:

CVaR	conditional value at risk
EF	emission factor
FAOSTAT	Food and Agriculture Organization Corporate Statistical Database
FPCM	fat- and protein-corrected milk
GLE	FAOSTAT Emissions from Livestock
GLEAM	Global Livestock Environmental Assessment Model
GWP	global warming potential
IPCC	Intergovernmental Panel on Climate Change
IQR	interquartile range
PAS	animals producing milk
QCL	FAOSTAT Crops and Livestock Products
TS	total stock
UNFCCC	United Nations Framework Convention on Climate Change
