# Supplementary material for: Ruminant Species Composition and Reported Methane Intensity in Global Milk Production: A 186-Country Decomposition Under Alternative Allocation Boundaries
Source: Animals (Basel). 2026 Oct 17;16(20):3273. doi: 10.3390/ani16203273 (PMC13700626; doi:10.3390/ani16203273)
Supplement: Supplementary file 1 [file animals-16-03273-s001.zip › Document S1-animals-4595225-supplementary.pdf]

Supplementary Materials

Ruminant Species Composition and Reported Methane Intensity in Global Milk Production: A 186-Country Decomposition Under Alternative Allocation Boundaries

Otto Ketney

Department of Agricultural Science and Food Engineering, Lucian Blaga University of Sibiu, 7–9 Dr. I. Ratiu Street, 550012 Sibiu, Romania; otto.ketney@ulbsibiu.ro

This document accompanies the article. Section S1 documents the re-extraction of the FAOSTAT data; S2 lists the series excluded from the analytical panel; S3 gives the Tier 1 diagnostics; S4 gives country-level results; S5 collects sensitivity analyses; S6 and S7 describe two analysis layers of the preprint version of this analysis (a hierarchical Bayesian smoother and a mean–conditional value-at-risk (CVaR) optimisation) that are not part of the present analysis, together with their re-assessment; S8 covers data and reproducibility. Numbers in this document are consistent with those of the main text.

S1. Re-Extraction of the FAOSTAT Data

The earlier database query (Google BigQuery) was not part of the public repository. It is replaced by an extraction made directly from the public FAOSTAT bulk files of the QCL domain (Production_Crops_Livestock_E_All_Data_(Normalized)) and the GLE domain (Emissions_livestock_E_All_Data_(Normalized)) at https://bulks-faostat.fao.org/production/, which yields one long table (country × species × year) with the values, flags and notes of each component. The items and elements are those of Table 1 of the main text.

Specification: (i) GLE rows with source 'FAO TIER 1' only (the 2020 values of some countries are duplicated by rows sourced from the United Nations Framework Convention on Climate Change (UNFCCC), which were discarded; the later years contain only the FAO Tier 1 series); (ii) regional aggregates (area code ≥ 5000), the 'China' aggregate (area code 351, M49 159) and 'Sub-Saharan Africa' (area code 420) were removed; (iii) methane is the sum of the enteric (72254) and manure-management (72256) elements, without global-warming-potential conversion; (iv) species rows without positive milk were dropped. The intensity is named explicitly (ch4_g_per_kg_raw_milk_whole_herd and ch4_g_per_kg_raw_milk_milk_allocated) in the analytical panel provided as Supplementary File S1.

Reconciliation with the earlier extraction. The new extraction contains 1,696 country–species–year cells with positive milk. Of these, 1,617 coincide with cells of the earlier extraction (1,637 rows before removal of the China aggregate); in every one of them the milk and the methane values are identical to the last reported digit (maximum relative difference in methane 3×10^{-16}; milk 0). The remaining 79 cells comprise 56 cells of five reporting entities that were missing from the earlier extraction (Saudi Arabia, the Syrian Arab Republic, the United Arab Emirates, the Central African Republic and South Africa) and 23 cells with milk but without GLE methane or counts in some years (Belgium, Cyprus, Luxembourg, Mongolian buffalo, the Netherlands, Slovakia and Venezuelan buffalo), which the complete-case rule excludes (Table S1).

S2. Analytical Panel and Excluded Series

Table S1. Country–species series excluded from the analytical panel (complete-case rule, Section 2.3 of the main text).

Country	Species	Years with milk	Milk 2023 (t)	Reason for exclusion from the complete-case panel
Belgium	goats	4	57,000	no GLE methane for 3 years; no QCL total-stock count; no milk-animal count
Belgium	sheep	1	370	milk series incomplete in 2020–2023; no GLE methane for 1 year; no QCL total-stock count; no milk-animal count
Cyprus	goats	4	37,280	no GLE methane for 1 year
Cyprus	sheep	4	49,620	no GLE methane for 1 year
Czechia	goats	1	0	milk series incomplete in 2020–2023; no milk-animal count
Czechia	sheep	1	0	milk series incomplete in 2020–2023; no milk-animal count
Estonia	goats	4	500	no milk-animal count
Germany	buffalo	1	0	milk series incomplete in 2020–2023; no milk-animal count
Germany	goats	1	0	milk series incomplete in 2020–2023; no milk-animal count
Germany	sheep	1	0	milk series incomplete in 2020–2023
Greece	buffalo	1	0	milk series incomplete in 2020–2023; no milk-animal count
Italy	buffalo	4	254,280	no milk-animal count
Luxembourg	goats	4	2,200	no GLE methane for 3 years; no QCL total-stock count; no milk-animal count
Luxembourg	sheep	4	70	no GLE methane for 1 year; no milk-animal count
Mongolia	buffalo	4	14,074	no GLE methane for 4 years; no QCL total-stock count
Netherlands (Kingdom of the)	buffalo	4	4,490	no GLE methane for 2 years; no QCL total-stock count; no milk-animal count; zero methane against positive milk
Palestine	cattle	3	0	milk series incomplete in 2020–2023
Palestine	goats	3	0	milk series incomplete in 2020–2023
Palestine	sheep	3	0	milk series incomplete in 2020–2023
Poland	goats	4	9,200	no milk-animal count
Poland	sheep	4	440	no milk-animal count
Romania	buffalo	4	19,300	no milk-animal count
Slovakia	goats	4	250	no GLE methane for 3 years; no QCL total-stock count; no milk-animal count
Slovakia	sheep	4	12,210	no milk-animal count
Venezuela (Bolivarian Republic of)	buffalo	4	433,008	no GLE methane for 4 years

A series is retained only if the required variables are positive in each of 2020–2023: milk, methane, milk animals and total stock for buffalo, goats, sheep and camels; milk and dairy-cattle methane for cattle (δ = 1, so milk animals and stock are not required). The 'buffalo' item of Mongolia (14,074 t of milk in 2023, 0.58 million animals in milk) has no GLE methane, and Mongolia has no

domestic buffalo herd; it most likely records the milk of other bovids (for example yak) under this item, and it is excluded.

S3. Tier 1 Diagnostics

The regression of ln I on ln(N/M) (whole-herd, N = GLE stock of the emission category) or ln(PAS/M) (milk-allocated) is described in Section 2.2 of the main text. The slope is 1 by construction when the emission factor is constant (the regression is therefore a check of the Tier 1 identity and not an independent result); departures arise from regional variation of the factor (cattle, partly sheep). The implied emission factor E/N varies by less than 0.010% within the series of 90% of the country–species pairs, which confirms that methane in the GLE domain is head count times a fixed factor.

Table S2. Regression of ln intensity on ln(N/M) (ln(PAS/M) for the milk-allocated boundary) by species and boundary, 2020–2023.

Species	Boundary	n	Countries	Slope	95% CI	R^2	EF median	EF IQR	Var ln EF	Var of predictor
cattle	whole-herd	740	185	0.808	0.758–0.856	0.891	73.00	47.00–110.00	0.177	1.517
buffalo	whole-herd	68	17	0.987	0.974–0.997	1.000	59.00	57.00–60.00	0.001	1.315
buffalo	milk-allocated	68	17	0.994	0.968–1.032	0.996	59.00	57.00–60.00	0.001	0.211
goats	whole-herd	412	103	1.001	1.000–1.002	1.000	5.17	5.13–5.18	0.000	2.296
goats	milk-allocated	412	103	1.002	0.999–1.006	0.998	5.17	5.13–5.18	0.000	1.110
sheep	whole-herd	284	71	0.943	0.906–0.982	0.977	5.20	5.15–8.19	0.045	1.861
sheep	milk-allocated	284	71	0.908	0.813–0.967	0.960	5.20	5.15–8.19	0.045	1.082
camels	whole-herd	116	29	0.999	0.997–1.002	1.000	47.98	47.92–48.56	0.000	2.127
camels	milk-allocated	116	29	0.986	0.955–0.999	0.982	47.98	47.92–48.56	0.000	1.084

EF, implied emission factor (kg CH_4 $head^{-1}$ $year^{-1}$), computed as GLE methane over GLE stock. 95% confidence interval (CI) from a country-clustered bootstrap (500 resamples). Var, variance across cells.

S4. Country-Level Results

Table S3. National methane (kt CH_4) and methane intensity (g CH_4 kg^{-1} raw milk) in 2023 under the whole-herd and the milk-allocated boundary, all countries of the analytical panel.

Country	Region	Species in the panel	Milk (kt)	CH_4 whole-herd (kt)	CH_4 milk-allocated (kt)	Intensity, whole-herd	Intensity, milk-allocated
Afghanistan	Asia	camel, cattle, goats, sheep	2,219.5	345.13	281.58	155.5	126.9
Albania	Europe	cattle, goats, sheep	927.6	47.41	45.34	51.1	48.9
Algeria	Africa	camel, cattle, goats, sheep	3,313.1	256.99	167.77	77.6	50.6
Angola	Africa	cattle	220.6	20.98	20.98	95.1	95.1
Antigua and Barbuda	Americas	cattle	2.9	0.17	0.17	60.6	60.6
Argentina	Americas	cattle	11,665.4	112.85	112.85	9.7	9.7

Country	Region	Species in the panel	Milk (kt)	CH_4 whole-herd (kt)	CH_4 milk-allocated (kt)	Intensity, whole-herd	Intensity, milk-allocated
Armenia	Asia	cattle, goats, sheep	591.6	14.23	13.25	24.1	22.4
Australia	Oceania	cattle	8,467.0	163.32	163.32	19.3	19.3
Austria	Europe	cattle, goats, sheep	3,717.0	78.64	75.49	21.2	20.3
Azerbaijan	Asia	cattle, goats, sheep	2,284.0	97.88	63.96	42.9	28.0
Bahamas	Americas	cattle, goats	2.1	0.13	0.09	62.6	42.7
Bahrain	Asia	cattle, goats, sheep	11.0	0.66	0.25	60.3	22.8
Bangladesh	Asia	buffalo, cattle, goats, sheep	14,068.0	798.87	635.87	56.8	45.2
Barbados	Americas	cattle	4.1	0.21	0.21	52.1	52.1
Belarus	Europe	cattle, goats	8,347.3	159.04	158.94	19.1	19.0
Belgium	Europe	cattle	4,627.0	73.91	73.91	16.0	16.0
Belize	Americas	cattle	6.5	0.44	0.44	67.7	67.7
Benin	Africa	cattle, goats	175.0	46.33	29.28	264.7	167.3
Bhutan	Asia	cattle	43.8	3.52	3.52	80.3	80.3
Bolivia (Plurinational State of)	Americas	cattle, goats, sheep	637.3	67.01	25.38	105.1	39.8
Bosnia and Herzegovina	Europe	cattle, sheep	583.4	27.90	20.05	47.8	34.4
Botswana	Africa	cattle, goats	34.2	6.22	0.69	182.1	20.2
Brazil	Americas	cattle, goats	36,338.7	1,209.78	1,143.76	33.3	31.5
Brunei Darussalam	Asia	buffalo, cattle	0.2	0.07	0.01	386.0	83.6
Bulgaria	Europe	buffalo, cattle, goats, sheep	815.8	33.29	30.18	40.8	37.0
Burkina Faso	Africa	camel, cattle, goats, sheep	440.9	195.90	98.13	444.3	222.6
Burundi	Africa	cattle, goats, sheep	97.7	27.71	8.53	283.5	87.3
Cabo Verde	Africa	cattle, goats	10.4	1.12	0.72	107.1	68.8
Cambodia	Asia	cattle	24.3	11.35	11.35	466.6	466.6
Cameroon	Africa	cattle, goats, sheep	283.1	67.14	25.65	237.1	90.6
Canada	Americas	cattle	9,886.6	169.77	169.77	17.2	17.2
Central African Republic	Africa	cattle	82.5	15.79	15.79	191.5	191.5
Chad	Africa	camel, cattle, goats, sheep	469.3	1,091.28	93.19	2,325.4	198.6
Chile	Americas	cattle, goats	2,232.4	41.88	40.99	18.8	18.4
China, Hong Kong SAR	Asia	cattle	0.1	0.00	0.00	31.7	31.7
China, Taiwan Province of	Asia	cattle, goats	483.6	6.43	6.06	13.3	12.5
China, mainland	Asia	buffalo, camel, cattle, goats, sheep	46,780.2	4,680.85	1,869.27	100.1	40.0
Colombia	Americas	cattle	7,309.0	257.15	257.15	35.2	35.2
Comoros	Africa	cattle	13.1	0.46	0.46	35.1	35.1
Congo	Africa	cattle	4.5	0.16	0.16	35.7	35.7
Costa Rica	Americas	cattle, goats	1,390.7	59.47	59.41	42.8	42.7
Croatia	Europe	cattle, goats, sheep	479.0	14.80	10.78	30.9	22.5
Cuba	Americas	cattle, goats	233.6	26.16	23.10	112.0	98.9

Country	Region	Species in the panel	Milk (kt)	CH_4 whole-herd (kt)	CH_4 milk-allocated (kt)	Intensity, whole-herd	Intensity, milk-allocated
Cyprus†	Asia	cattle	303.9	1.86	1.86	6.1	6.1
Czechia	Europe	cattle	3,475.3	39.34	39.34	11.3	11.3
Côte d'Ivoire	Africa	cattle	37.8	15.35	15.35	406.0	406.0
Democratic People's Republic of Korea	Asia	cattle	84.2	2.78	2.78	33.0	33.0
Democratic Republic of the Congo	Africa	cattle	9.8	0.53	0.53	53.6	53.6
Denmark	Europe	cattle	5,687.0	75.46	75.46	13.3	13.3
Djibouti	Africa	camel, cattle	15.3	4.73	2.73	309.2	178.5
Dominica	Americas	cattle	7.0	0.55	0.55	78.3	78.3
Dominican Republic	Americas	cattle	862.2	41.34	41.34	47.9	47.9
Ecuador	Americas	cattle, goats, sheep	2,105.3	64.47	62.11	30.6	29.5
Egypt	Africa	buffalo, cattle, goats, sheep	5,633.7	172.42	123.75	30.6	22.0
El Salvador	Americas	cattle	417.0	26.02	26.02	62.4	62.4
Eritrea	Africa	camel, cattle, goats, sheep	147.8	76.25	39.80	515.9	269.3
Estonia	Europe	cattle	894.2	11.50	11.50	12.9	12.9
Eswatini	Africa	cattle	40.6	5.58	5.58	137.6	137.6
Ethiopia	Africa	camel, cattle, goats, sheep	3,783.8	1,007.62	480.72	266.3	127.0
Faroe Islands	Europe	cattle	7.0	0.15	0.15	21.2	21.2
Fiji	Oceania	cattle	12.7	5.50	5.50	432.2	432.2
Finland	Europe	cattle	2,239.0	32.61	32.61	14.6	14.6
France	Europe	cattle, goats, sheep	25,050.5	500.60	457.57	20.0	18.3
French Polynesia	Oceania	cattle	0.5	0.05	0.05	99.1	99.1
Gabon	Africa	cattle	10.7	0.39	0.39	36.0	36.0
Gambia	Africa	cattle	51.9	1.75	1.75	33.8	33.8
Georgia	Asia	cattle, goats, sheep	601.8	25.37	21.59	42.2	35.9
Germany	Europe	cattle	34,012.6	512.37	512.37	15.1	15.1
Ghana	Africa	cattle	50.5	18.30	18.30	362.6	362.6
Greece	Europe	cattle, goats, sheep	1,962.5	91.76	69.61	46.8	35.5
Grenada	Americas	cattle	0.6	0.06	0.06	101.0	101.0
Guatemala	Americas	cattle, goats	562.3	53.34	52.82	94.9	93.9
Guinea	Africa	cattle, goats, sheep	293.1	86.12	47.94	293.9	163.6
Guinea-Bissau	Africa	cattle, goats, sheep	38.9	12.01	6.39	308.8	164.3
Guyana	Americas	cattle	28.6	2.89	2.89	100.9	100.9
Haiti	Americas	cattle, goats	100.2	23.82	17.67	237.7	176.4
Honduras	Americas	cattle	693.1	37.36	37.36	53.9	53.9
Hungary	Europe	cattle, goats, sheep	2,032.1	36.90	29.58	18.2	14.6

Country	Region	Species in the panel	Milk (kt)	CH_4 whole-herd (kt)	CH_4 milk-allocated (kt)	Intensity, whole-herd	Intensity, milk-allocated
Iceland	Europe	cattle	156.0	3.97	3.97	25.4	25.4
India	Asia	buffalo, camel, cattle, goats	239,306.9	11,537.46	7,079.43	48.2	29.6
Indonesia	Asia	buffalo, cattle, goats, sheep	1,373.1	293.69	128.81	213.9	93.8
Iran (Islamic Republic of)	Asia	buffalo, cattle, goats, sheep	8,477.2	498.76	223.74	58.8	26.4
Iraq	Asia	buffalo, camel, cattle, goats, sheep	363.0	84.98	45.65	234.1	125.8
Ireland	Europe	cattle	8,730.7	208.50	208.50	23.9	23.9
Israel†	Asia	cattle, goats, sheep	1,687.3	8.73	7.20	5.2	4.3
Italy	Europe	cattle, goats, sheep	13,587.9	315.91	298.69	23.2	22.0
Jamaica	Americas	cattle, goats	208.7	3.36	3.17	16.1	15.2
Japan	Asia	cattle	7,298.9	65.21	65.21	8.9	8.9
Jordan	Asia	cattle, goats, sheep	642.7	21.27	16.49	33.1	25.7
Kazakhstan	Asia	camel, cattle, goats	3,439.9	124.83	108.43	36.3	31.5
Kenya	Africa	camel, cattle, goats, sheep	6,068.1	741.46	350.10	122.2	57.7
Kuwait	Asia	cattle, goats, sheep	58.8	5.41	0.69	92.1	11.7
Kyrgyzstan	Asia	cattle, goats	1,778.3	72.86	71.02	41.0	39.9
Lao People's Democratic Republic	Asia	cattle	9.1	4.64	4.64	511.5	511.5
Latvia	Europe	cattle, goats	963.7	16.48	16.46	17.1	17.1
Lebanon	Asia	cattle, goats, sheep	222.6	7.83	6.10	35.2	27.4
Lesotho	Africa	cattle	225.0	5.85	5.85	26.0	26.0
Liberia	Africa	cattle	8.3	0.32	0.32	38.0	38.0
Libya	Africa	camel, cattle, goats, sheep	226.7	61.71	20.05	272.2	88.4
Lithuania	Europe	cattle, goats	1,473.0	29.32	29.30	19.9	19.9
Luxembourg	Europe	cattle	469.3	7.62	7.62	16.2	16.2
Madagascar	Africa	cattle	439.1	68.08	68.08	155.1	155.1
Malawi	Africa	cattle, goats, sheep	224.5	74.19	20.28	330.5	90.3
Malaysia	Asia	buffalo, cattle	46.3	8.14	4.25	175.8	91.8
Mali	Africa	camel, cattle, goats, sheep	1,114.1	457.05	341.01	410.2	306.1
Malta	Europe	cattle, goats, sheep	40.4	1.11	1.09	27.5	27.0
Mauritania	Africa	camel, cattle, goats, sheep	362.5	191.83	45.99	529.3	126.9
Mauritius	Africa	cattle	2.8	0.14	0.14	51.2	51.2
Mexico	Americas	cattle, goats	13,909.7	243.79	202.06	17.5	14.5
Mongolia	Asia	camel, cattle, goats, sheep	250.5	395.76	202.66	1,579.6	808.9
Montenegro	Europe	cattle, goats, sheep	161.4	8.33	7.42	51.6	46.0
Morocco	Africa	camel, cattle, goats, sheep	2,046.6	212.95	89.01	104.0	43.5
Mozambique	Africa	cattle, goats	23.0	21.38	1.20	927.7	52.2
Myanmar	Asia	buffalo, cattle, goats, sheep	2,242.4	251.85	133.61	112.3	59.6

Country	Region	Species in the panel	Milk (kt)	CH_4 whole-herd (kt)	CH_4 milk-allocated (kt)	Intensity, whole-herd	Intensity, milk-allocated
Namibia	Africa	cattle	113.6	13.67	13.67	120.3	120.3
Nepal	Asia	buffalo, cattle, goats, sheep	2,770.7	316.39	120.99	114.2	43.7
Netherlands (Kingdom of the)	Europe	cattle, goats	15,112.6	217.08	216.87	14.4	14.4
New Caledonia	Oceania	cattle	0.1	0.04	0.04	380.0	380.0
New Zealand	Oceania	cattle	21,245.0	524.28	524.28	24.7	24.7
Nicaragua	Americas	cattle	1,372.9	74.35	74.35	54.2	54.2
Niger	Africa	camel, cattle, goats, sheep	1,641.8	391.25	188.96	238.3	115.1
Nigeria	Africa	cattle	526.5	104.78	104.78	199.0	199.0
Niue	Oceania	cattle	0.1	0.01	0.01	154.6	154.6
North Macedonia	Europe	cattle, goats, sheep	291.7	16.16	14.44	55.4	49.5
Norway	Europe	cattle, goats	1,409.4	28.56	28.36	20.3	20.1
Oman	Asia	cattle, goats, sheep	376.1	23.39	17.33	62.2	46.1
Pakistan	Asia	buffalo, camel, cattle, goats, sheep	64,581.6	4,388.75	2,102.52	68.0	32.6
Panama	Americas	cattle	184.3	10.66	10.66	57.8	57.8
Papua New Guinea	Oceania	cattle, goats	0.2	0.21	0.20	1,035.1	990.3
Paraguay	Americas	cattle	559.0	15.33	15.33	27.4	27.4
Peru	Americas	cattle, goats	2,221.5	77.26	69.44	34.8	31.3
Philippines	Asia	cattle	30.0	1.24	1.24	41.4	41.4
Poland	Europe	cattle	15,482.2	246.77	246.77	15.9	15.9
Portugal	Europe	cattle, goats, sheep	2,058.7	53.21	36.55	25.8	17.8
Puerto Rico	Americas	cattle, goats	223.9	4.39	4.38	19.6	19.6
Qatar	Asia	camel, cattle, goats, sheep	44.0	11.31	3.41	256.8	77.5
Republic of Korea†	Asia	cattle, goats	1,938.9	16.71	15.43	8.6	8.0
Republic of Moldova	Europe	cattle, goats, sheep	261.3	11.03	10.03	42.2	38.4
Romania	Europe	cattle, goats, sheep	4,299.1	207.88	186.30	48.4	43.3
Russian Federation	Europe	camel, cattle, goats, sheep	33,804.7	870.78	737.68	25.8	21.8
Rwanda	Africa	cattle, goats, sheep	235.5	26.13	20.03	111.0	85.0
Saint Lucia	Americas	cattle	1.0	0.05	0.05	52.9	52.9
Saint Vincent and the Grenadines	Americas	cattle	0.9	0.04	0.04	44.5	44.5
Samoa	Oceania	cattle	1.6	0.20	0.20	125.0	125.0
Sao Tome and Principe	Africa	cattle	0.6	0.02	0.02	34.3	34.3
Saudi Arabia	Asia	camel, cattle, goats, sheep	3,280.3	234.74	44.95	71.6	13.7
Senegal	Africa	camel, cattle, goats, sheep	257.4	110.03	42.14	427.4	163.7
Serbia	Europe	cattle, goats, sheep	1,416.8	63.91	50.12	45.1	35.4
Seychelles	Africa	cattle	0.2	0.01	0.01	73.4	73.4
Sierra Leone	Africa	cattle	150.6	5.53	5.53	36.7	36.7

Country	Region	Species in the panel	Milk (kt)	CH_4 whole-herd (kt)	CH_4 milk-allocated (kt)	Intensity, whole-herd	Intensity, milk-allocated
Slovakia	Europe	cattle	913.6	12.64	12.64	13.8	13.8
Slovenia	Europe	cattle, goats, sheep	607.8	14.16	13.13	23.3	21.6
Solomon Islands	Oceania	cattle	3.1	0.28	0.28	90.3	90.3
Somalia	Africa	camel, cattle, goats, sheep	2,163.5	532.98	235.86	246.4	109.0
South Africa	Africa	cattle	3,810.0	47.07	47.07	12.4	12.4
South Sudan	Africa	cattle, goats, sheep	3,518.9	565.66	479.71	160.7	136.3
Spain	Europe	cattle, goats, sheep	8,564.6	236.52	138.53	27.6	16.2
Sri Lanka	Asia	buffalo, cattle, goats	535.3	43.35	29.21	81.0	54.6
Sudan	Africa	camel, cattle, goats, sheep	4,511.5	978.28	668.22	216.8	148.1
Suriname	Americas	cattle	2.8	0.07	0.07	26.6	26.6
Sweden	Europe	cattle	2,818.5	39.88	39.88	14.1	14.1
Switzerland	Europe	cattle, goats, sheep	3,767.2	75.70	72.61	20.1	19.3
Syrian Arab Republic	Asia	buffalo, cattle, goats, sheep	2,066.2	129.97	94.94	62.9	45.9
Tajikistan	Asia	cattle, goats	1,175.5	101.49	91.91	86.3	78.2
Thailand	Asia	cattle	1,278.3	26.61	26.61	20.8	20.8
Timor-Leste	Asia	goats	5.5	0.88	0.22	161.3	39.4
Togo	Africa	cattle	12.8	3.12	3.12	243.0	243.0
Tonga	Oceania	cattle	0.3	0.02	0.02	76.6	76.6
Trinidad and Tobago	Americas	cattle	1.4	0.53	0.53	368.7	368.7
Tunisia	Africa	camel, cattle, goats, sheep	1,422.5	69.04	23.09	48.5	16.2
Turkmenistan	Asia	camel, cattle, goats	2,609.2	120.64	100.30	46.2	38.4
Türkiye	Asia	buffalo, cattle, goats, sheep	21,481.6	611.61	467.68	28.5	21.8
Uganda	Africa	cattle	3,850.0	183.74	183.74	47.7	47.7
Ukraine	Europe	cattle, goats, sheep	7,430.4	152.40	149.62	20.5	20.1
United Arab Emirates	Asia	camel, cattle, goats, sheep	266.5	53.12	25.79	199.3	96.8
United Kingdom of Great Britain and Northern Ireland	Europe	cattle	15,759.5	256.89	256.89	16.3	16.3
United Republic of Tanzania	Africa	cattle, goats	4,317.6	569.11	452.56	131.8	104.8
United States of America	Americas	cattle, goats	102,678.8	1,666.92	1,655.26	16.2	16.1
Uruguay	Americas	cattle	2,177.4	50.59	50.59	23.2	23.2
Uzbekistan	Asia	cattle	11,944.0	448.42	448.42	37.5	37.5
Vanuatu	Oceania	cattle	3.1	1.23	1.23	395.8	395.8
Venezuela (Bolivarian Republic of)	Americas	cattle, goats	3,737.2	219.07	214.51	58.6	57.4
Viet Nam	Asia	buffalo, cattle	1,246.3	151.64	31.34	121.7	25.1
Yemen	Asia	camel, cattle, goats, sheep	434.2	147.77	44.16	340.4	101.7
Zambia	Africa	cattle	542.7	18.06	18.06	33.3	33.3

Country	Region	Species in the panel	Milk (kt)	CH_4 whole-herd (kt)	CH_4 milk-allocated (kt)	Intensity, whole-herd	Intensity, milk-allocated
Zimbabwe	Africa	cattle	443.1	48.33	48.33	109.1	109.1

Species-level values for every country, species and year are in the analytical panel (Supplementary File S1). Intensity = $10^6 \times CH_4$ / milk. † Israel, Cyprus and the Republic of Korea: the low values follow from fixed regional Tier 1 factors applied to very high reported yields (Israel, Republic of Korea) or from cattle-only coverage (Cyprus, whose goat and sheep methane is not published by GLE in 2023); Cyprus is assigned to Asia by the United Nations M49 regions (Western Asia).

Table S4. Country-level exact Shapley decomposition of the 2020–2023 change in national intensity (g CH_4 kg^{-1} raw milk).

Country	Weight (kt)	Whole-herd structure	Whole-herd within	Whole-herd total	Milk-alloc. structure	Milk-alloc. within	Milk-alloc. total
Afghanistan	2,205	−0.90	+3.56	2.65	−0.20	+3.55	3.36
Albania	1,006	0.08	−3.04	−2.95	0.04	−1.50	−1.46
Algeria	3,334	−2.18	+5.79	3.61	−1.41	+3.96	2.55
Angola	217	0.00	−2.35	−2.35	0.00	−2.35	−2.35
Antigua and Barbuda	3	0.00	−5.63	−5.63	0.00	−5.63	−5.63
Argentina	11,556	0.00	−0.19	−0.19	0.00	−0.19	−0.19
Armenia	623	−1.05	+1.48	0.43	−0.66	+0.95	0.28
Australia	8,783	0.00	+1.06	1.06	0.00	+1.06	1.06
Austria	3,785	0.04	+1.36	1.40	0.00	+1.38	1.37
Azerbaijan	2,238	−1.17	−2.78	−3.95	−0.05	−0.91	−0.96
Bahamas	2	−0.03	+0.35	0.32	−0.09	+0.38	0.29
Bahrain	11	−0.72	+1.63	0.91	0.00	+1.37	1.37
Bangladesh	12,374	0.00	−16.57	−16.57	0.00	−12.37	−12.37
Barbados	4	0.00	−1.90	−1.90	0.00	−1.90	−1.90
Belarus	8,056	0.00	−1.99	−1.99	0.00	−1.98	−1.98
Belgium	4,542	0.00	−0.68	−0.68	0.00	−0.68	−0.68
Belize	6	0.00	−31.20	−31.20	0.00	−31.20	−31.20
Benin	152	−9.86	−12.43	−22.29	5.46	−23.57	−18.11
Bhutan	51	0.00	−3.47	−3.47	0.00	−3.47	−3.47
Bolivia (Plurinational State of)	629	−2.64	−0.41	−3.05	−0.37	+0.18	−0.20
Bosnia and Herzegovina	614	−29.23	+19.75	−9.47	−1.72	−11.79	−13.51
Botswana	35	−85.21	+80.12	−5.09	−5.98	+6.90	0.92
Brazil	36,373	0.11	−0.53	−0.42	0.00	−0.53	−0.53
Brunei Darussalam	0	−9.31	−206.42	−215.72	3.23	−8.19	−4.96
Bulgaria	911	−1.39	+2.48	1.09	−0.98	+1.40	0.43
Burkina Faso	431	−0.33	−22.05	−22.38	1.97	−9.23	−7.26
Burundi	103	45.91	+12.98	58.89	−9.82	+12.32	2.50
Cabo Verde	10	0.84	0.00	0.84	−0.45	−0.37	−0.82
Cambodia	24	0.00	−27.11	−27.11	0.00	−27.11	−27.11
Cameroon	274	−8.10	−8.35	−16.45	−0.70	−6.27	−6.97
Canada	9,764	0.00	−0.73	−0.73	0.00	−0.73	−0.73
Central African Republic	82	0.00	+5.93	5.93	0.00	+5.93	5.93
Chad	446	13.41	+186.99	200.40	−0.21	+4.81	4.60
Chile	2,292	0.04	+0.91	0.95	0.02	+0.94	0.96
China, Hong Kong SAR	0	0.00	0.00	0.00	0.00	0.00	0.00
China, Taiwan Province of	467	−0.23	−0.40	−0.64	−0.02	−0.31	−0.34
China, mainland	42,833	−9.56	+1.37	−8.19	−1.19	+0.19	−1.00
Colombia	7,190	0.00	−0.92	−0.92	0.00	−0.92	−0.92
Comoros	13	0.00	−0.03	−0.03	0.00	−0.03	−0.03
Congo	4	0.00	−0.34	−0.34	0.00	−0.34	−0.34
Costa Rica	1,304	0.02	−7.83	−7.81	0.02	−7.85	−7.83
Croatia	546	−0.89	−2.87	−3.76	−0.13	−4.26	−4.40
Cuba	346	3.65	+50.73	54.38	−0.18	+49.26	49.08
Cyprus	290	0.00	−0.78	−0.78	0.00	−0.78	−0.78
Czechia	3,372	0.00	−0.70	−0.70	0.00	−0.70	−0.70
Côte d'Ivoire	36	0.00	−26.18	−26.18	0.00	−26.18	−26.18
Democratic People's Republic of Korea	85	0.00	+0.04	0.04	0.00	+0.04	0.04
Democratic Republic of the Congo	9	0.00	+0.90	0.90	0.00	+0.90	0.90

Country	Weight (kt)	Whole-herd structure	Whole-herd within	Whole-herd total	Milk-alloc. structure	Milk-alloc. within	Milk-alloc. total
Denmark	5,676	0.00	−0.49	−0.49	0.00	−0.49	−0.49
Djibouti	15	−0.38	−1.36	−1.74	−0.10	−0.84	−0.93
Dominica	7	0.00	+0.18	0.18	0.00	+0.18	0.18
Dominican Republic	877	0.00	+2.09	2.09	0.00	+2.09	2.09
Ecuador	2,213	0.21	−1.01	−0.80	0.04	−1.08	−1.04
Egypt	5,606	−1.07	+1.98	0.91	−0.37	+0.71	0.35
El Salvador	414	0.00	−1.16	−1.16	0.00	−1.16	−1.16
Eritrea	148	1.93	−0.24	1.69	−0.15	+0.03	−0.12
Estonia	871	0.00	−0.85	−0.85	0.00	−0.85	−0.85
Eswatini	39	0.00	−18.93	−18.93	0.00	−18.93	−18.93
Ethiopia	3,792	−11.54	+35.10	23.55	−0.12	+23.56	23.45
Faroe Islands	7	0.00	+0.27	0.27	0.00	+0.27	0.27
Fiji	13	0.00	+116.34	116.34	0.00	+116.34	116.34
Finland	2,323	0.00	−0.10	−0.10	0.00	−0.10	−0.10
France	25,670	0.05	−0.54	−0.49	−0.01	−0.45	−0.46
French Polynesia	1	0.00	+29.26	29.26	0.00	+29.26	29.26
Gabon	11	0.00	−0.16	−0.16	0.00	−0.16	−0.16
Gambia	51	0.00	−2.04	−2.04	0.00	−2.04	−2.04
Georgia	594	−0.65	−1.15	−1.79	−0.08	−1.70	−1.78
Germany	33,589	0.00	−1.25	−1.25	0.00	−1.25	−1.25
Ghana	49	0.00	+5.07	5.07	0.00	+5.07	5.07
Greece	1,976	0.20	+0.07	0.27	0.14	−1.48	−1.34
Grenada	1	0.00	+6.91	6.91	0.00	+6.91	6.91
Guatemala	562	0.00	+3.03	3.04	0.00	+3.03	3.03
Guinea	277	−0.64	+0.26	−0.38	0.07	−10.49	−10.42
Guinea-Bissau	39	−0.11	+3.14	3.03	−0.09	+0.67	0.59
Guyana	25	0.00	−36.49	−36.49	0.00	−36.49	−36.49
Haiti	99	−1.61	+3.97	2.36	−3.09	+1.85	−1.24
Honduras	689	0.00	+0.28	0.28	0.00	+0.28	0.28
Hungary	2,026	−0.03	+0.75	0.73	0.00	+0.93	0.93
Iceland	156	0.00	+2.58	2.58	0.00	+2.58	2.58
India	224,637	−0.39	−3.77	−4.17	0.07	−1.30	−1.23
Indonesia	1,466	9.13	+7.20	16.33	1.72	+7.95	9.66
Iran (Islamic Republic of)	8,411	0.11	+3.03	3.13	−0.12	−0.76	−0.88
Iraq	352	0.06	−1.03	−0.97	−1.51	−2.77	−4.28
Ireland	8,646	0.00	+0.41	0.41	0.00	+0.41	0.41
Israel	1,671	0.07	−0.39	−0.32	0.01	−0.31	−0.30
Italy	13,422	−0.19	−1.44	−1.63	−0.12	−1.33	−1.45
Jamaica	206	−0.15	−0.37	−0.53	−0.16	−0.41	−0.57
Japan	7,369	0.00	+0.14	0.14	0.00	+0.14	0.14
Jordan	526	0.73	−20.00	−19.28	0.23	−14.32	−14.09
Kazakhstan	3,301	0.02	−0.20	−0.18	−0.02	−0.02	−0.03
Kenya	5,838	−9.26	−7.78	−17.04	−0.92	−5.91	−6.83
Kuwait	58	−0.71	+0.89	0.18	−0.01	+0.51	0.49
Kyrgyzstan	1,723	−0.07	−0.78	−0.85	−0.03	−0.42	−0.45
Lao People's Democratic Republic	9	0.00	+9.54	9.54	0.00	+9.54	9.54
Latvia	977	−0.01	−1.91	−1.92	0.00	−1.92	−1.92
Lebanon	262	−3.55	+12.79	9.23	−1.95	+10.50	8.54
Lesotho	179	0.00	−17.56	−17.56	0.00	−17.56	−17.56
Liberia	8	0.00	−0.40	−0.40	0.00	−0.40	−0.40
Libya	228	6.27	+2.74	9.01	1.21	+0.37	1.58
Lithuania	1,482	0.00	−1.69	−1.69	0.00	−1.69	−1.68
Luxembourg	458	0.00	−0.49	−0.49	0.00	−0.49	−0.49
Madagascar	448	0.00	−1.07	−1.07	0.00	−1.07	−1.07
Malawi	213	3.08	+9.20	12.28	−0.21	+0.15	−0.06
Malaysia	47	7.31	+12.60	19.91	−0.71	+8.92	8.21
Mali	1,055	4.60	−1.19	3.41	3.73	−0.39	3.34
Malta	43	0.29	+2.30	2.59	0.21	+2.27	2.47
Mauritania	359	−0.53	+1.83	1.29	−0.39	+0.34	−0.05
Mauritius	3	0.00	−6.46	−6.46	0.00	−6.46	−6.46
Mexico	13,509	−0.08	−0.39	−0.47	0.00	−0.29	−0.29
Mongolia	220	−15,190.97	+14,580.69	−610.28	−5,833.42	+5,514.87	−318.56
Montenegro	174	−1.73	+4.52	2.79	−0.49	+2.46	1.98
Morocco	2,382	15.27	+3.71	18.98	1.36	+5.71	7.07
Mozambique	22	23.34	+90.63	113.97	−0.25	+4.62	4.38
Myanmar	2,170	−0.73	−3.94	−4.67	0.04	−4.06	−4.02
Namibia	107	0.00	+16.84	16.84	0.00	+16.84	16.84
Nepal	2,611	−7.92	−62.80	−70.72	0.53	−31.25	−30.72
Netherlands (Kingdom of the)	15,021	−0.01	−0.32	−0.33	−0.01	−0.32	−0.33

Country	Weight (kt)	Whole-herd structure	Whole-herd within	Whole-herd total	Milk-alloc. structure	Milk-alloc. within	Milk-alloc. total
New Caledonia	0	0.00	+303.55	303.55	0.00	+303.55	303.55
New Zealand	21,558	0.00	−0.39	−0.39	0.00	−0.39	−0.39
Nicaragua	1,386	0.00	−0.22	−0.22	0.00	−0.22	−0.22
Niger	1,576	−6.95	−1.84	−8.79	0.60	−5.45	−4.85
Nigeria	523	0.00	+0.26	0.26	0.00	+0.26	0.26
Niue	0	0.00	−0.42	−0.42	0.00	−0.42	−0.42
North Macedonia	376	6.06	+3.81	9.87	3.43	+4.03	7.45
Norway	1,489	0.00	+1.26	1.26	0.00	+1.22	1.23
Oman	363	−1.10	+0.16	−0.94	−0.58	−0.25	−0.83
Pakistan	61,642	−0.41	+0.03	−0.39	0.10	0.00	0.09
Panama	199	0.00	+8.37	8.37	0.00	+8.37	8.37
Papua New Guinea	0	−0.87	+0.01	−0.86	−1.12	+0.24	−0.89
Paraguay	550	0.00	−1.57	−1.57	0.00	−1.57	−1.57
Peru	2,190	−0.15	−0.05	−0.20	−0.01	0.00	−0.01
Philippines	29	0.00	−0.24	−0.24	0.00	−0.24	−0.24
Poland	15,152	0.00	+0.16	0.16	0.00	+0.16	0.16
Portugal	2,079	−0.35	−0.58	−0.93	−0.05	−0.82	−0.87
Puerto Rico	233	0.01	−0.15	−0.14	0.00	−0.15	−0.15
Qatar	47	−5.32	−23.13	−28.44	−0.98	−3.25	−4.23
Republic of Korea	2,018	0.06	+0.22	0.29	0.01	+0.24	0.25
Republic of Moldova	292	1.62	+1.92	3.53	1.14	+1.64	2.78
Romania	4,323	1.01	−2.32	−1.31	0.72	−1.93	−1.21
Russian Federation	33,012	0.50	−2.74	−2.25	0.10	−1.78	−1.68
Rwanda	245	−0.59	−4.76	−5.35	0.07	−2.03	−1.96
Saint Lucia	1	0.00	−0.23	−0.23	0.00	−0.23	−0.23
Saint Vincent and the Grenadines	1	0.00	+0.40	0.40	0.00	+0.40	0.40
Samoa	2	0.00	+16.51	16.51	0.00	+16.51	16.51
Sao Tome and Principe	1	0.00	−1.42	−1.42	0.00	−1.42	−1.42
Saudi Arabia	3,093	−6.04	+8.86	2.81	−0.89	−2.54	−3.43
Senegal	252	2.04	−29.35	−27.31	0.02	−26.11	−26.09
Serbia	1,500	2.78	−3.90	−1.12	0.16	−2.37	−2.22
Seychelles	0	0.00	−1.55	−1.55	0.00	−1.55	−1.55
Sierra Leone	147	0.00	+0.06	0.06	0.00	+0.06	0.06
Slovakia	916	0.00	−0.80	−0.80	0.00	−0.80	−0.80
Slovenia	620	0.72	−0.73	−0.01	0.02	−0.13	−0.11
Solomon Islands	3	0.00	−0.31	−0.31	0.00	−0.31	−0.31
Somalia	2,187	2.89	+0.83	3.72	0.77	+0.28	1.05
South Africa	3,832	0.00	+0.14	0.14	0.00	+0.14	0.14
South Sudan	3,325	−2.69	−17.28	−19.98	1.43	−16.50	−15.08
Spain	8,625	−0.50	−1.48	−1.98	−0.06	−0.58	−0.64
Sri Lanka	529	4.36	−5.22	−0.86	0.53	−0.47	0.06
Sudan	4,583	0.11	+0.50	0.61	0.04	+0.73	0.77
Suriname	3	0.00	+2.05	2.05	0.00	+2.05	2.05
Sweden	2,796	0.00	−1.00	−1.00	0.00	−1.00	−1.00
Switzerland	3,788	0.05	−0.37	−0.32	0.00	−0.37	−0.37
Syrian Arab Republic	2,110	3.36	+4.67	8.03	2.04	+3.92	5.96
Tajikistan	1,125	−0.13	−12.05	−12.17	0.09	−12.14	−12.05
Thailand	1,298	0.00	+0.83	0.83	0.00	+0.83	0.83
Timor-Leste	6	0.00	−2.02	−2.02	0.00	+1.07	1.07
Togo	12	0.00	−19.78	−19.78	0.00	−19.78	−19.78
Tonga	0	0.00	−0.23	−0.23	0.00	−0.23	−0.23
Trinidad and Tobago	2	0.00	+123.24	123.24	0.00	+123.24	123.24
Tunisia	1,418	1.67	−3.08	−1.41	0.03	−0.72	−0.70
Turkmenistan	2,537	−2.32	−1.59	−3.91	−0.85	−3.14	−3.99
Türkiye	22,493	−0.78	+1.62	0.84	−0.34	+0.89	0.56
Uganda	3,316	0.00	−17.77	−17.77	0.00	−17.77	−17.77
Ukraine	8,347	−0.01	−1.33	−1.34	0.00	−1.30	−1.30
United Arab Emirates	262	0.96	+5.72	6.68	0.49	−9.91	−9.42
United Kingdom of Great Britain and Northern Ireland	15,723	0.00	−0.03	−0.03	0.00	−0.03	−0.03
United Republic of Tanzania	3,877	−4.01	−12.61	−16.62	0.14	−14.00	−13.86
United States of America	101,998	0.00	−0.12	−0.13	0.00	−0.12	−0.12
Uruguay	2,224	0.00	−0.84	−0.84	0.00	−0.84	−0.84
Uzbekistan	11,437	0.00	0.00	0.00	0.00	0.00	0.00
Vanuatu	3	0.00	−121.98	−121.98	0.00	−121.98	−121.98
Venezuela (Bolivarian Republic of)	3,411	1.07	−24.27	−23.20	1.41	−23.85	−22.45
Viet Nam	1,177	−17.81	−8.33	−26.14	−0.11	−3.92	−4.03
Yemen	411	−1.14	−24.86	−26.00	0.05	−12.05	−12.00
Zambia	490	0.00	−2.57	−2.57	0.00	−2.57	−2.57

Country	Weight (kt)	Whole-herd structure	Whole-herd within	Whole-herd total	Milk-alloc. structure	Milk-alloc. within	Milk-alloc. total
Zimbabwe	427	0.00	+0.98	0.98	0.00	+0.98	0.98

Weight, mean of 2020 and 2023 national milk output. Structure + within = total change in national intensity, exactly.

S5. Sensitivity Analyses

Table S5. World aggregate ratio of the analytical panel (g CH_4 kg^{-1} raw milk).

Year	World ratio, whole-herd	World ratio, milk-allocated	Milk in panel (Mt)
2020	47.38	30.38	918.7
2021	46.82	30.01	941.5
2022	46.72	29.89	952.1
2023	46.30	29.74	970.2

Table S6. Number of countries per species in the balanced analytical panel.

Species	2020	2021	2022	2023
cattle	185	185	185	185
buffalo	17	17	17	17
goats	103	103	103	103
sheep	71	71	71	71
camels	29	29	29	29

Table S7. All influence and sensitivity cases of the decomposition (g CH_4 kg^{-1} raw milk).

Case	Boundary	n	Structure	Within	Net	Δ world ratio	Country shares	Species mix	Intensity
full panel	whole	186	−4.243	+1.883	−2.360	−1.078	+1.524	−4.354	+1.752
Mongolia sheep series excluded	whole	186	−0.976	−1.367	−2.342	−1.067	+1.285	−0.981	−1.372
Mongolia excluded	whole	185	−0.713	−1.506	−2.219	−1.035	+1.180	−0.711	−1.503
five largest structural contributors excluded	whole	181	−0.116	−1.081	−1.197	−0.285	+0.909	−0.115	−1.079
ten largest structural contributors excluded	whole	176	−0.047	−0.981	−1.028	−0.277	+0.750	−0.046	−0.981
denominator-collapse cells screened (series removed)	whole	186	−0.976	−1.367	−2.342	−1.067	+1.285	−0.981	−1.372
full panel	milk	186	−1.383	+0.264	−1.119	−0.641	+0.573	−1.427	+0.213
Mongolia sheep series excluded	milk	186	−0.097	−1.018	−1.115	−0.635	+0.483	−0.099	−1.019
Mongolia excluded	milk	185	−0.027	−1.018	−1.045	−0.618	+0.426	−0.027	−1.016
five largest structural contributors excluded	milk	181	0.019	−1.176	−1.156	−0.674	+0.481	0.019	−1.175
ten largest structural contributors excluded	milk	176	0.013	−0.953	−0.939	−0.565	+0.373	0.013	−0.952
denominator-collapse cells screened (series removed)	milk	186	−0.097	−1.018	−1.115	−0.635	+0.483	−0.099	−1.019
milk-allocated, GLE stock as TS	milk	186	−1.380	+0.312	−1.069	−0.589	+0.576	−1.425	+0.261
milk-allocated, GLE stock as TS, Mongolia sheep excluded	milk	186	−0.094	−0.971	−1.065	−0.582	+0.486	−0.097	−0.972
milk-allocated, cattle delta = min(1, PAS / GLE dairy stock)	milk	186	−1.385	+0.277	−1.108	−0.642	+0.562	−1.429	+0.226
milk-allocated, cattle delta = min(1, PAS / GLE dairy stock), Mongolia sheep excluded	milk	186	−0.098	−1.006	−1.104	−0.635	+0.473	−0.101	−1.007

Case	Boundary	n	Structure	Within	Net	Δ world ratio	Country shares	Species mix	Intensity
FPCM functional unit (milk-allocated)	milk	186	−1.161	+0.209	−0.952	−0.646	+0.370	−1.192	+0.175
FPCM functional unit, Mongolia sheep excluded	milk	186	−0.019	−0.941	−0.960	−0.641	+0.323	−0.022	−0.942

The rows 'GLE stock as TS' use the stock of the GLE domain instead of the QCL live-animal stock in the allocation factor. FPCM, fat- and protein-corrected milk.

Table S8. Leave-one-country-out analysis of the production-weighted structural term (g CH_4 kg^{-1} raw milk).

Boundary	Minimum structure (any country removed except Mongolia)	Country	Maximum	Country	Structure without Mongolia	Within without Mongolia
Whole-herd	−5.44	India	−3.99	China, mainland	−0.71	−1.51
Milk-allocated	−1.84	India	−1.38	Burundi	−0.03	−1.02

Each country was removed in turn and the decomposition recomputed.

Reconciliation of the Mongolian milk series with national statistics. Total milk reported by the National Statistics Office of Mongolia [1] was compared with the sum of the five FAOSTAT milk items of Mongolia, and the species-level milk per animal in milk was derived from the FAOSTAT milk and milk-animal counts (the table below). The two totals coincide to within 0.1 t in 2020–2022 and differ by 0.6% in 2023. The FAOSTAT stocks of sheep, goats and camels equal the national census counts of the National Statistics Office [1] in every year of 2020–2023 (the GLE stock of cattle refers to the dairy category and is not comparable with the census count of all cattle). In 2023 the census counts are 29.41 million sheep and 24.62 million goats, 10.2% and 10.7% below 2022, and the census count of cattle falls by 2.9%, from 5.51 to 5.35 million head. The species split of milk is not published by the National Statistics Office in its public tables (national and aimag totals only), so the abrupt change of the split in 2023 could not be reconciled at source.

Table S9. Mongolia: national milk total and species-level FAOSTAT milk, 2020–2023.

Year	NSO total milk (t)	FAOSTAT five items (t)	Difference (t)	Cattle milk (t)	Goat milk (t)	Sheep milk (t)	Cattle, t per milk animal	Goats, t per milk animal	Sheep, t per milk animal
2020	200,166	200,165	0	125,878	40,811	20,335	0.083	0.0041	0.00166
2021	186,576	186,576	0	122,054	34,494	17,663	0.085	0.0042	0.00158
2022	220,739	220,739	0	139,309	43,712	21,617	0.082	0.0042	0.00167
2023	266,254	264,609	−1,644	235,527	13,491	572	0.142	0.0016	0.00005

NSO, National Statistics Office of Mongolia (data.1212.mn, retrieved 2 October 2026; tables DT_NSO_1001_041V1 and DT_NSO_1001_021V1). The five FAOSTAT items are cattle, goat, sheep, camel and buffalo raw milk; the buffalo item equals the residual between the national total and the four other species in 2020–2022.

Table S10. Sensitivity of the reported intensity to a bounded compositional counterfactual: all budgets and cases.

Boundary	Case	b	Production-weighted counterfactual decrease (%)	Unweighted mean counterfactual decrease, all 186 (%)	Mean counterfactual decrease, eligible (%)	Median counterfactual decrease, eligible (%)	IQR, eligible	Cattle lowest	Donor shares exhausted
whole	all data	1%	7.0	4.8	8.4	3.8	1.4–9.8	89	17
whole	all data	5%	16.9	10.4	18.2	14.5	5.2–23.1	89	32
whole	all data	10%	23.4	13.7	23.8	17.5	7.4–32.4	89	42
whole	all data	20%	27.9	17.0	29.6	21.4	10.9–43.8	89	56
whole	Mongolia sheep excluded	10%	23.2	13.6	23.7	17.5	7.4–32.4	89	42
milk	all data	1%	1.5	1.3	2.3	1.3	0.7–2.3	53	12
milk	all data	5%	4.6	4.0	6.9	4.7	2.5–8.1	53	25
milk	all data	10%	7.2	6.2	10.7	7.3	4.1–14.2	53	33
milk	all data	20%	10.0	9.0	15.6	12.7	6.3–21.1	53	40
milk	Mongolia sheep excluded	10%	7.0	6.1	10.5	7.3	4.1–14.2	53	33
milk	FPCM	10%	7.0	5.6	9.7	7.6	4.0–12.2	52	28

The unweighted mean over all 186 countries includes the structural zeros of single-species countries and gives equal weight to very small producers; it is reported for comparison with the preprint version only. Eligible, countries with at least two species (n = 107).

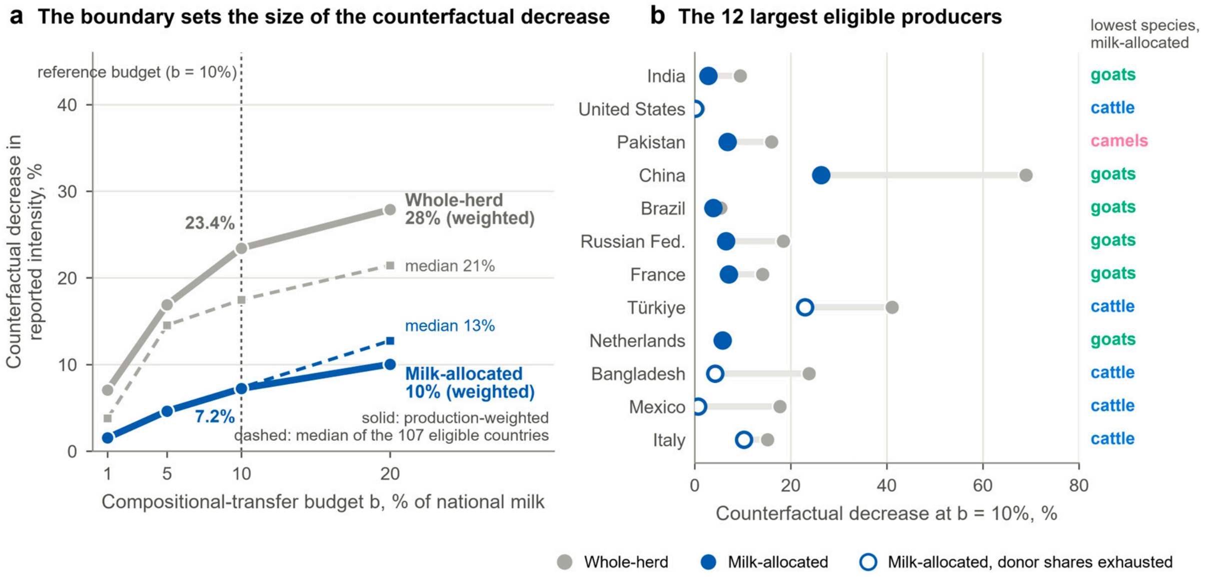

Figure S1. Sensitivity of the reported intensity to a bounded compositional counterfactual. **(a)** Counterfactual decrease in the reported intensity as a function of the compositional-transfer budget b (% of national milk), production-weighted (solid) and median of the 107 eligible countries (dashed). **(b)** Counterfactual decrease at b = 10% in the 12 largest eligible producers under the whole-herd (grey) and milk-allocated (blue) boundary; open blue symbols mark countries in which the transfer exhausts the

represented milk shares of all higher-intensity donor species, and the lowest-intensity species under milk allocation is printed at the right.

Sensitivity to the length of the study window. The main analysis covers 2020–2023, a recent interval chosen to describe the most recent four years of the Tier 1 inventory; the QCL and GLE series start in 1961, so the window is a design choice and not a limit of the data. Table S11 repeats the three-factor decomposition of the change in the world aggregate ratio for windows beginning in 2015 and 2010 (end year 2023), with the same indicators, complete-case rule and denominator-collapse screening. Over longer windows the world ratio falls more. In these windows, under milk allocation the decline is carried by the within-species term and the species-mix term stays small (at most 0.6 g CH_4 kg^{-1} in absolute value); under the whole-herd boundary species mix contributes more, but within-species change remains the largest component. The attribution of the main text is therefore not specific to the four-year window.

Table S11. Sensitivity of the aggregate decomposition to the length of the study window (three-factor decomposition of the change in the world aggregate ratio, g CH_4 kg^{-1} raw milk).

Window	Boundary	Case	Countries	World ratio, start → end	Change	Country shares	Species mix	Within-species
2020–2023	milk-allocated	full panel	186	30.38 → 29.74	−0.64	+0.57	−1.43	+0.21
2020–2023	milk-allocated	flagged series removed	186	30.31 → 29.68	−0.64	+0.48	−0.10	−1.02
2020–2023	whole-herd	full panel	186	47.38 → 46.30	−1.08	+1.52	−4.35	+1.75
2020–2023	whole-herd	flagged series removed	186	47.21 → 46.14	−1.07	+1.29	−0.98	−1.37
2015–2023	milk-allocated	full panel	186	33.38 → 29.74	−3.64	+0.99	−0.58	−4.05
2015–2023	milk-allocated	flagged series removed	186	33.32 → 29.68	−3.64	+0.95	−0.03	−4.56
2015–2023	whole-herd	full panel	186	51.54 → 46.28	−5.26	+2.71	−3.21	−4.76
2015–2023	whole-herd	flagged series removed	186	51.38 → 46.12	−5.26	+2.60	−1.83	−6.03
2010–2023	milk-allocated	full panel	183	35.31 → 28.79	−6.52	+2.15	−0.03	−8.64
2010–2023	milk-allocated	flagged series removed	183	35.24 → 28.70	−6.54	+2.17	−0.06	−8.65
2010–2023	whole-herd	full panel	183	54.45 → 45.06	−9.39	+4.67	−2.33	−11.73
2010–2023	whole-herd	flagged series removed	183	54.12 → 44.74	−9.38	+4.78	−2.83	−11.32

Window: first and last year; the end year is 2023 in all cases. A country–species series enters only if it is complete in every year of the window (186, 186 and 183 countries; 99.9%, 99.8% and 99.0% of the 2023 raw milk). Flagged series (denominator-collapse rule, Section 2.3 of the main text): Mongolian sheep milk in all windows and, in 2010–2023, also Russian sheep milk. The 2020–2023 rows reproduce Table 4 of the main text. Script 12_longer_window.py.

S6. Hierarchical Smoother of the Preprint Version (Superseded)

This analysis is retained solely for audit of the preprint version of this analysis and is not used in any result or conclusion of the article.

The preprint version fitted a hierarchical Bayesian Student-*t* model to log species intensities, $\ln I_{c,s,t} \sim \text{Student-}t_{\nu}(\mu_{c,s,t}, \sigma_s)$ with $\mu_{c,s,t} = \alpha_s + u_c + \beta_s(t - 2020) + \gamma_s \cdot 1[t \geq 2022]$, and used its posterior as the reference intensity of the optimisation. The model was estimated by the No-U-Turn Sampler (NUTS; 16 chains, 15,000 tuning and 8,000 posterior iterations; PyMC 5.16.2) on the 181-country frozen panel. It was not re-estimated for the present analysis, which it does not enter; the results quoted here are those of the preprint run.

Why it is not used in the present analysis. (i) The country effect u_c is shared by all species, so for two species in the same country and year the country effect cancels and the ratio of their latent intensities is identical in every country; the within-country species ordering is imposed by the specification and cannot reproduce national orderings such as that of Greek dairy sheep. (ii) The dispersion parameter τ did not converge (potential scale reduction factor $\hat{R} = 1.19$, bulk effective sample size (ESS) = 65), although the identified product u_c did. (iii) The priors were centred on summaries of the same outcome. (iv) The posterior reference differs strongly from the observed ratio in the countries that carry the scenario result: the median ratio of the posterior to the observed national intensity is 0.99, but only 66% of the 181 countries lie within ±25% of the observed value, and 3 of the 20 largest producers lie outside it (Table S12). Because intensities are now computed from observed country-specific values, none of these issues affects the present results. The smoother is retained here for audit only; because it was not re-estimated on the present panel, it cannot serve as a check of the present results.

Table S12. Posterior reference intensity of the smoother of the preprint version against the observed national intensity (g CH_4 kg^{-1}) for the 20 largest milk producers of the preprint panel.

Country	Milk (Mt)	Observed	Posterior	Posterior/observed
India	239.3	48.2	79.9	1.66
United States of America	102.7	16.2	16.1	0.99
Pakistan	64.6	68.0	137.5	2.02
China, mainland	46.8	100.1	46.1	0.46
Brazil	36.3	33.3	31.8	0.96
Germany	34.0	15.1	16.0	1.06
Russian Federation	33.8	25.8	21.9	0.85
France	25.1	20.0	22.6	1.13
Türkiye	21.5	28.5	26.4	0.93
New Zealand	21.2	24.7	24.7	1.00
United Kingdom of Great Britain and Northern Ireland	15.8	16.3	16.2	1.00
Poland	15.5	16.1	15.3	0.95
Netherlands (Kingdom of the)	15.1	14.4	15.9	1.11
Bangladesh	14.1	56.8	62.7	1.10
Mexico	13.9	17.5	14.9	0.85
Italy	13.8	24.6	29.2	1.19
Uzbekistan	11.9	37.5	37.3	0.99
Argentina	11.7	9.7	9.6	0.99
Canada	9.9	17.2	17.4	1.01

Country	Milk (Mt)	Observed	Posterior	Posterior/observed
Ireland	8.7	23.9	23.1	0.97

Of the 20 countries with the largest scenario reductions of Table 3 of the preprint version, 12 had posterior/observed ratios outside ±25% and carried 80% of the absolute total of that table; the 20 largest milk producers of the preprint panel are shown here, of which 3 lie outside that range. The two selections are different subsets of the same posterior.

S7. Mean–CVaR Optimisation of the Preprint Version (Superseded)

This analysis is retained solely for audit of the preprint version of this analysis and is not used in any result or conclusion of the article.

The preprint version solved, for each country, a linear program that minimised a convex combination of the posterior mean and the conditional value at risk of national intensity under a reallocation budget (Rockafellar–Uryasev formulation, HiGHS solver) [2,3]. In the preprint run the risk term did not change the selected composition in 180 of the 181 countries. With intensities fixed at their observed values the problem is deterministic and its optimum is the closed form of Equation (8) of the main text. We verified the closed form against a general-purpose solver (HiGHS linear-programming solver) for all 186 countries and both boundaries: the largest difference between the objective values is 5×10^{-13} g CH_4 kg^{-1}. The 36-cell parameter grid, the Dirichlet perturbation of the shares and the 'do-no-harm' guard of the preprint analysis are therefore not needed to obtain the reported quantity, and the layer is replaced by the bounded compositional counterfactual of Section 2.6.

S8. Data and Reproducibility

The analytical panel (Supplementary File S1; country × species × year, with milk, milk animals, total stock, methane, the allocation factor δ, both intensities and the FAOSTAT flags and notes of each component) and its data dictionary allow every result of the main text and of this document to be recomputed. The extraction script (01_extract_faostat.py), the analysis code (02_analysis.py), the window sensitivity of Table S11 (12_longer_window.py) and all result tables are deposited, with SHA-256 checksums, in the public GitHub repository https://github.com/ketney1982/global-milk-emissions-inequality (tag v4.0.1-R1, commit 7545379; accessed on 15 October 2026), and the reproduction commands are given in its README. No global-warming-potential conversion is applied anywhere, and all intensities are expressed in g CH_4 kg^{-1} raw milk.

Statement on pre-specification. The decision rules for influence analysis (leave-one-out, removal of the largest contributors, denominator-collapse rule) are described in Section 2.3. No analysis protocol was deposited before the analysis, and the manuscript makes no claim that the rules were defined in advance.

References

1. National Statistics Office of Mongolia. Production of agriculture, by type, region, aimags and the Capital (DT_NSO_1001_041V1); Number of livestock (DT_NSO_1001_021V1). Available online: https://data.1212.mn (accessed on 2 October 2026).
2. Rockafellar, R.T.; Uryasev, S. Optimization of conditional value-at-risk. *J. Risk* **2000**, *2*, 21–41, doi:10.21314/JOR.2000.038.
3. Huangfu, Q.; Hall, J.A.J. Parallelizing the dual revised simplex method. *Math. Program. Comput.* **2018**, *10*, 119–142, doi:10.1007/s12532-017-0130-5.
